# Effects of Vegetation Strips, Fertilizer Levels and Varietal Resistance on the Integrated Management of Arthropod Biodiversity in a Tropical Rice Ecosystem

**DOI:** 10.3390/insects10100328

**Published:** 2019-10-01

**Authors:** Finbarr G. Horgan, Eduardo Crisol Martínez, Alexander M. Stuart, Carmencita C. Bernal, Elena de Cima Martín, Maria Liberty P. Almazan, Angelee Fame Ramal

**Affiliations:** 1 EcoLaVerna Integral Restoration Ecology, Bridestown, Kildinan, T56CD39, Co. Cork, Ireland; eduardocrisol@googlemail.com; 2 COEXPHAL (Association of Vegetable and Fruit Growers of Almeria), C\Esteban Murillo, 3, 04746 La Mojonera, Almeria, Spain; 3 International Rice Research Institute, DAPO Box 7777, Metro Manila 1301, Philippines; a.stuart@irri.org (A.M.S.); c.bernal@irri.org (C.C.B.); eledecima@gmail.com (E.d.C.M.); m.l.almazan@irri.org (M.L.P.A.); 4 School of Environmental Science and Management, University of the Philippines, 4030 Los Baños, Laguna, Philippines; angelee.ramal@gmail.com

**Keywords:** agroecology, ecological engineering, IPM, Philippines, key rice pests

## Abstract

Integrated biodiversity management aims to conserve the beneficial species components of production ecosystems and reduce the impacts of pests. In 2011 and 2013, experiments were conducted at Los Baños, Laguna, Philippines, to compare arthropod communities in rice plots and on levees with and without vegetation strips. Vegetation strips included spontaneous weeds, sesame and okra (2011), or mung bean (2013). The plots were treated with one of three nitrogen levels and in one experiment were planted with planthopper-resistant (IR62) and susceptible (IR64) rice varieties. Parasitoids and predators of lepidopteran pests and of the ricebug, *Leptocorisa oratorius*, were more abundant in high-nitrogen rice plots where their prey/hosts also had highest densities. Planthoppers and leafhoppers were more abundant in low-nitrogen plots. Weedy and sesame/okra bunds provided habitat for a range of natural enemies including spiders, parasitoids and predatory bugs, but did not have higher pest numbers than cleared bunds. Higher abundances of the predator *Cythorhinus* *lividipennis* and higher parasitism of planthopper (*Nilaparvata lugens*) eggs by *Anagrus* sp. were associated with sesame/okra bunds in late season rice plots. Mung bean also provided habitat for key predators and parasitoids that spilled over to adjacent rice; however, mung bean was also associated with higher numbers of lepidopteran and grain-sucking pests in the adjacent rice, albeit without increased damage to the rice. For ricebug in particular, damage was probably reduced by higher parasitoid:pest ratios adjacent to the vegetation strips. Varietal resistance and mung bean strips had an additive effect in reducing abundance of the planthopper *Sogatella furcifera* and the leafhopper *Nephotettix virescens*. Reduced numbers of these latter pests close to vegetation strips were often compensated for by other plant-sucking bugs, thereby increasing the intensity of potentially stabilizing interspecific interactions such as competition. We highlight the benefits of diversifying rice landscapes and the need to optimize vegetation strips, e.g., by including lepidopteran trap-plants, for intensive rice production systems.

## 1. Introduction

Cropland, with its associated remnant natural vegetation, is a major anthropogenic biome throughout much of South and South East Asia [1]. In Asia’s lowland coastal regions, rice production landscapes dominate this biome. Rice is the principal staple food in tropical Asia and is produced on an estimated 108 M ha in South and South East Asia [2,3]. Over 80% of this rice area lies in lowland coastal regions. Following the global financial and food crises of 2008, there has been increasing pressure on Asian farmers to intensify production methods to attain food security targets, particularly in rice [4,5,6]. However, to ensure further economic growth, the types of intensification practices promoted among farmers have been frequently driven by government and institutional partnerships with industry [6,7]. This, together with phenomenal industrial growth in China and India since the early 2000s, has created an environment where agrochemicals are increasingly overused or misused. For example, since 2002, pesticide trade in Asia has increased by over 340% [2,8]. Intensification of rice production has resulted in increased rice harvests over much of Asia; however, for many nations, the rate of yield increase in relation to inputs has declined in recent decades [8]. This overuse of fertilizers and pesticides has several negative consequences. These include contamination of food, soils and water that affects human and wildlife health, a reduced profitability of farming, and a loss of ecosystem services such as the regulation by natural enemies of agricultural pests [8,9,10,11,12]. Indeed, the intensification of rice production has been associated with increased outbreaks of several pest species, including planthoppers (Hemiptera: Delphacidae), leaffolders (Lepidoptera: Pyralidae) and stemborers (Lepidoptera: Crambidae, Noctuidae, Pyralidae). Each of these pests benefits from the higher nutrient availability of chemically fertilized food plants, and from the enemy-free space created by broad-spectrum insecticides [10,12,13].

Realization of the negative effects of fertilizers and pesticides, and the role of enemy-free space in initiating outbreaks of rice pests, has recently placed biodiversity management at the forefront of public research into rice crop protection. Over the last decade, research institutes in several Asian countries have initiated and/or completed projects aimed at enhancing regulation services provided by the natural enemies of rice pests, particularly planthopper and stemborer pests (e.g., China, Thailand and Vietnam [14]; the Philippines: [5]; Indonesia [15], India: [16]; and Bangladesh: [17]). The results from many of these projects have been generally positive. For example, comparative studies have indicated that planting rice levees (known as bunds) with strips or patches of nectar-producing flowers increases natural enemy abundance and/or reduces pest incidence [14,16,17,18,19]. This can lead to a reduction in insecticide applications [14,20] and increased rice yields [14,17]. Planting vegetables on bunds also provides a supplementary income for rice farmers and promotes diet diversification of the farming household [18,20,21]. Diversifying rice fields can also increase the quality of habitat for vertebrates such as wild birds and amphibians [18,20]. In contrast, the use of standard practice insecticide applications in these studies frequently resulted in increased insect damage [14,17,21,22]. Despite such positive results, this type of research continues to receive considerably less institutional support than other pest management approaches such as the use of biocides or host plant resistance [8]. Furthermore, researchers interested in pest management have tended to focus only on their chosen management approach with little attention to integrating advances in targeted pest management, such as pheromone technologies or host plant resistance, with biodiversity management approaches, such as conservation biological control or ecological engineering [8,9].

Previous field studies have indicated how fertilizers [23,24,25], varieties [26,27], and vegetation strips [21,28,29] can each affect arthropod communities in rice ecosystems. There have been some studies on the comparative and combined [14,22] effects of vegetation strips and pesticides on rice arthropods, but less attention has been given to the integration of other production practices with vegetation strips for biodiversity management [30]. Whereas pesticides, can have destabilizing effects on pest populations through their negative impact on pest regulation by natural enemies, particularly when combined with high fertilizer levels [21,31,32,33,34], resistant varieties and vegetation strips are expected to promote stability by reducing pest population growth rates [9] and by enhancing pest regulation [14,17,19,21], respectively. Vegetation strips, and other agroecological approaches to pest management further aim to reduce the suitability and quality of the agroecosystem for pest populations while simultaneously creating conditions that enhance the diversity and abundance of natural enemies. Increasing the diversity of natural enemies in agroecosystems is predicted to bring greater stability to arthropod communities and populations [35,36,37,38], preventing pest outbreaks and countering the potential destabilizing effects of fertilizers, pesticides and climate change.

The present study examines the suitability of different types of vegetation strip as habitat for beneficial and pest arthropods. It assesses whether the positive or negative effects of these vegetation strips may spill over to adjacent rice habitat to counter the impacts of fertilizers that increase pest abundance. To our knowledge, no previous studies of the role of vegetation strips in rice ecosystems have incorporated fertilizer rates or varietal resistance into field designs, despite a large focus on both of these factors in recent literature concerning rice pest management [8,9,30]. In particular, we included host plant resistance as a factor in our experiments to assess possible additive effects in countering the impacts of high nitrogen. Due to restrictions on field space during the experiments, and because the main focus of the research was on the vegetation strips, relatively small-scale plots were used in the present study, with sampling restricted to within 3 m of the rice bunds. It is difficult to infer from the results of small plots such as these to larger field and landscape scales; however, we assume that the effects of vegetation strips diminish at increasing distances into the rice crop (i.e., away from the bunds). Indeed, this has been indicated in previous studies with rice [14,19]. Therefore, the design mainly helped in gaining practical knowledge of bund management that could be translated into guidelines for farmers interested in diversifying their rice fields. As such, the results of this study should be regarded as preliminary indicators for improving or amplifying current strategies for biodiversity management in rice. They indicate future avenues for practical field designs by eliminating potentially counterproductive interventions and by highlighting management approaches that deserve further research attention.

## 2. Materials and Methods

### 2.1. Experimental Platform

Field experiments were conducted during the 2011 and 2013 wet seasons (WS) at the International Rice Research Institute (IRRI) Experimental Field Station (Los Baños, Laguna, Philippines). Seeds of all rice varieties were acquired through the IRRI Germplasm Collection. Vegetable seeds were acquired through the Agronomy Faculty at the University of the Philippines, Los Baños. No pesticides were used in the fields at any time during the experiments.

**Figure 1:**
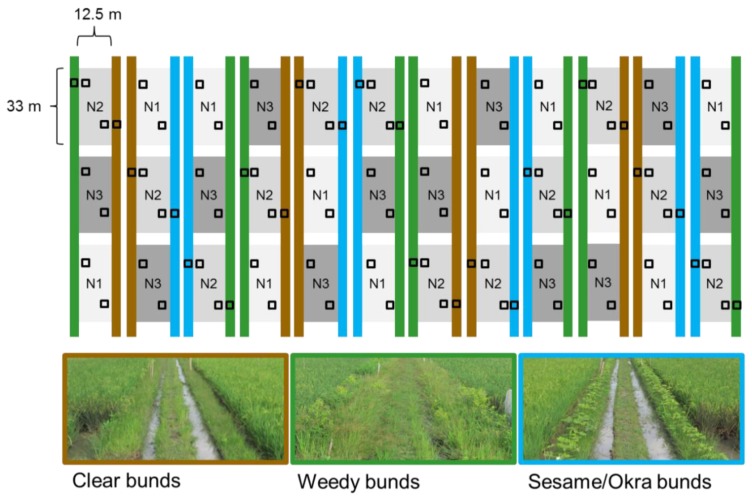
Field set-up during the 2011 wet season indicating fertilizer levels (N1 = 0 added nitrogen, N2 = 60 Kg ha^−1^ of added nitrogen, N3 = 150 Kg ha^−1^ of added nitrogen) and bund treatments (brown = clear, green = weedy, and blue = sesame/okra bunds). All fields were planted with IR66. Squares indicate approximate sampling points. Photographs indicate the appearance of bunds during rice crop growth. Note the central levees and sub-drainage channels between adjacent treatment bunds.

During the 2011 WS, 12 rice fields (encompassing 36 nitrogen treatment plots of 33 × 12.5 m (L × W)) were planted with IR66 (a variety reported to contain the *bph4* gene [39,40]). Field plots were treated with one of three nitrogen levels each season. These were: zero added nitrogen (0 Kg N ha^−1^), 60 Kg N ha^−1^ and 150 Kg N ha^−1^. Nitrogen (ammonium sulphate) was applied as four top dressings (basal, mid-tillering, panicle initiation and at one week before flowering). Solophos, muriate of potash and zinc were applied basally with the ammonium sulphate. Separate sub-irrigation channels were installed around each plot. These connected to the main field canals for irrigation and drainage but prevented leakage of nutrients between adjacent fields or between plots within each field. Seed was initially sown to dry seedbeds (9 May 2011) and the ‘seedlings’ (pre-tillering stage) were transplanted 28 days later (6 June 2011), as one plant per hill to the puddled field plots. Hills were spaced at a distance of 20 cm. The rice bunds were assigned ecological engineering or control treatments prior to rice transplanting. There were three bund management treatments: (1) Cleared bunds that were periodically mowed to keep weed vegetation below 15 cm; (2) weedy bunds that had natural weed cover without mowing; and, (3) sesame/okra bunds that were planted with a combination of sesame (*Sesamum indicum* L.) and okra (*Abelmoschus esculentus* [L.] Moench)—these were mowed at the start of the season to reduce weed competition. The bunds were then planted with sesame and okra seed at the time of rice transplanting (early June). We used sesame and okra on the bunds following a series of preliminary experiments by the authors to assess germination and seedling survival of a range of locally available vegetable crops on unmanaged rice bunds. Furthermore, sesame has been shown to promote the survival and development of several important natural enemies of rice planthoppers and stemborers [41,42]. Adjacent bunds of the same type were separated by at least an intermediate walkway levee and two sub-drainage channels. Figure 1 indicates the experimental set-up during the 2011 WS.

**Figure 2:**
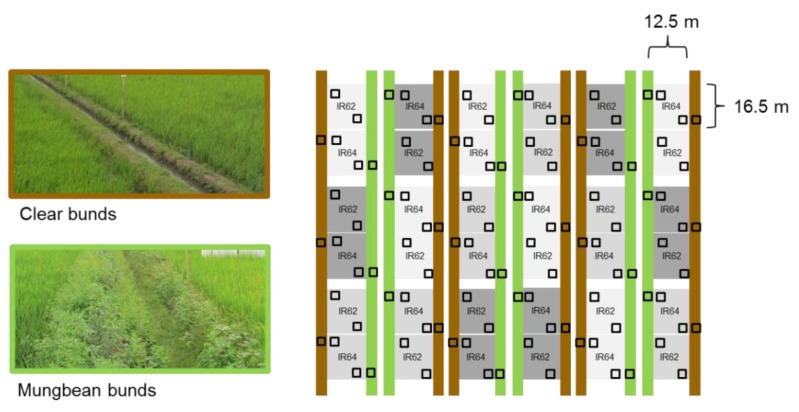
Field set-up during the 2013 wet season indicating fertilizer levels (light grey = 0 added nitrogen, medium grey = 60 Kg ha^−1^ of added nitrogen, dark grey = 150 Kg ha^−1^ of added nitrogen) and bund treatments (brown = clear bunds, light green = mung bean bunds). Nitrogen-treatment plots were subdivided into IR62 (resistant) and IR64 (susceptible/tolerant) rice varieties. Squares indicate approximate sampling points. Photographs indicate the appearance of bunds during rice crop growth. Note the more rapid growth of the mung bean plants on the left, high-nitrogen bund compared with the right, low-nitrogen bund in the lower photograph.

During the 2013 WS, six rice fields (18 plots) were planted with IR62 and IR64. IR62 has been noted for its high resistance to planthoppers and leafhoppers and the consistency of its resistance across Asia [43]. IR62 contains the *Bph3(t)* gene locus (possibly including the *Bph32* gene) for resistance to the brown planthopper, *Nilaparvata lugens* (Stål) (Hemiptera: Delphacidae) [44]. IR64 has been a popular variety grown by farmers since its release in 1985 [39]. The variety contains the *Bph1* gene as well as tolerance traits [45]. Experiments at IRRI indicated that *N. lugens* has adapted to survive on rice with the *Bph1* gene locus [43], such that the variety is considered susceptible to the planthopper throughout much of Asia. The two varieties were planted to the 18 nitrogen-treated plots as variety sub-plots (each 16.5 × 12.5 m). Field plots were treated with zero added nitrogen (0 Kg N ha^−1^), 60 Kg N ha^−1^ or 150 Kg N ha^−1^ with irrigation channels designed to prevent nutrient leakage as described for the 2011 WS experiment (above). Nitrogen (ammonium sulphate) was applied as four top dressings (basal, mid-tillering, panicle initiation and at one week before flowering). Solophos, muriate of potash and zinc were applied basally with the ammonium sulphate. Small plots such as these are considered adequate to assess yield and other plant characteristics in experiments such as ours where all plant genotypes used in the experiment have similar growth and development (i.e., short varieties with similar rates of maturation [46,47,48]). Seed was initially sown to dry seedbeds (3 June 2013) and the ‘seedlings’ transplanted at 28 days after sowing on 1 July 2013 as one plant per hill to the puddled field plots. Hills were spaced at 20 cm. The rice bunds were assigned ecological engineering or control treatments prior to transplanting. The ecological engineering bunds were sown with mung bean (*Vigna radiata* [L.] R. Wilczek) seeds one month before rice transplanting. Mung bean was used based on good germination and high seedling survival on rice bunds during preliminary investigations (see above, unpublished). The control bunds were cleared of weeds by mowing, as described above. Figure 2 indicates the experimental set-up during the 2013 WS.

### 2.2. Bund Development and Plot Yields

The growth and development of the rice crop and vegetation strips was monitored during both crop seasons. The bund vegetation was measured (height) and all vegetation cut above the roots at three randomly selected 1 m lengths of bund per plot, for each bund type (on 1 September 2011 and 2 September 2013 for the first and second experiments, respectively). The vegetation was dried in a forced draught oven for 1 week and then separated into weeds and vegetables. The weeds were identified in 2011, and weighed in both years. The yield of mung bean plants per 1m of bund was estimated in 2013. Vegetable yields were not estimated in 2011 because of outside interference and removal of fruits. Rice was harvested on 26 September 2011 and 11 October 2013 for the first and second field seasons, respectively, when grain maturity was ≥80%. IR66 had a duration of 112 days, and IR62 and IR64 had a duration of 103 days in the field. At harvest, two rice areas of 1 m^2^ were sampled for yield in each sub-plot, one adjacent to each of the different bund types.

### 2.3. Effects of Nitrogen and Bund Types on Arthropod Fauna

Rice in each field plot was sampled for arthropods at about 30 days after transplanting (DAT) and 60 DAT during 2011 and 2013, with a further sample prior to harvest during 2013 (sampling dates = 25 July and 24 August 2011; 1 August, 2 September and 10 October 2013). Sampling was conducted at 30 DAT to represent the mid-tillering (vegetative) phase of the rice crop and 60 DAT to represent the reproductive phase (mainly booting). Arthropods were sampled using sweep netting and Blow-vac suction sampling. These methods have been assessed for their accuracy in reflecting arthropod communities in rice fields [21,49,50,51,52]. Each method samples slightly different components of the arthropod fauna. Sweepnets capture greater numbers of large arthropods that inhabit the rice canopy, whereas Blow-vac sampling more efficiently captures micro-Hymenoptera such as egg parasitoids and arthropods that occur close to the water surface [21,49,50]. Neither method is suitable for sampling the eggs of arthropods and preimaginal stages of egg-parasitoids, or the larvae of stemborers and other pest species that occur inside the rice stem. We therefore also examined whole rice plants (method described in Horgan et al. [21]) and used sentinel trap plants (described below) to assess these arthropod life stages; however, because of low numbers of stemborer larvae and pest egg masses recovered during whole plant sampling, we do not report our results in the present paper.

Sweep netting was conducted using standard entomological sweep nets (diameter = 40 cm) with 10 sweeps across the height of the rice crop. In both years, samples from the rice plots were collected at between 1 and 3 m from the bunds (see Figure 1 and Figure 2). A total of 144 samples of arthropods from the rice crop were collected in 2011 (sampling period [2] × bund type [3] × nitrogen level [3] × variety [1] × replicates [8] = 144). Bunds were sampled for arthropods once, at 66 DAT (1 September), during 2011 using a Blow-vac suction sampler [51]. Samples were collected only adjacent to rice plots treated with 60 Kg N ha^−1^. There were 24 samples (sampling period [1] × bund type [3] × nitrogen level [1] × variety [1] × replicates [8] = 24). To enable Blow-vac sampling, a transparent acetate cage (100 × 60 × 60 cm, H × L × W) was first quickly placed over the vegetation to be sampled (bund vegetation) and covered with a fine mesh cloth to prevent any flying insects from escaping. All arthropods inside the cage were then sucked through the Blow-vac into glass vials.

In 2013, the bunds were sampled at the same time as the rice crop using sweepnets (2 September). Samples for each of the three temporal sampling points were collected only from the IR64 sub-plots and adjacent bunds. Sweepnet sampling of the bunds was conducted in 2013 instead of Blow-vac sampling (that was conducted in 2011) because of relatively low captures of arthropods during previous Blow-vac sampling. Also, standardization of sampling protocols for rice and bunds in 2013 allowed direct comparisons of the arthropods present in both habitat types during that year. Sweepnet sampling from the rice and bunds each consisted of 10 sweeps across the height of the rice plants in the field plots or the grasses and weeds on the clear bunds, and at the height of the mung bean plants in the vegetable strips. A total of 216 samples were collected to examine the effects of nitrogen and bund types in 2013 (sampling period [3] × bund type [2] × rice/bund [2] × nitrogen level [3] × variety [1] × replicates [6] = 216).

Samples were stored in 90% ethanol and all arthropod species were identified, maintaining a voucher collection of morphospecies. Arthropods sampled at preimaginal stages were identified using an extensive voucher collection available at IRRI. For rice pests and their natural enemies, arthropods were identified to genus or species level. For all other arthropods, specimens were identified to at least family level. Rice pests and natural enemies were determined according to Heinrichs [52] and Pathak and Khan [53].

### 2.4. Effects of Nitrogen, Variety and Bund Type on Planthoppers, Leafhoppers and Their Natural Enemies

In 2013, a further set of samples was collected from the IR62 and IR64 sub-plots to examine the combined effects of nitrogen, variety and bund type on the planthoppers and leafhoppers attacking rice plants, and on the abundance of their principal natural enemies. For these samples, we focused only on this subset of arthropods as these are the most likely groups to be affected by the resistance levels of the host plants. Samples were collected using sweep netting of the rice plants (as described above, Section 2.3). There were 72 samples (sampling period [1] × bund type [2] × nitrogen level [3] × variety [2] × replicates [6] = 72). Bunds were not sampled. Specimens were identified to species or genus level as described above, and were stored in 90% ethanol.

### 2.5. Herbivore Regulation

The potential for regulation of two pest species (*N. lugens* and the yellow stemborer, *Scirpophaga incertulas* (Walker) (Lepidoptera: Crambidae)) by egg predators and parasitoids was monitored at 30 and 60 DAT in 2011. To monitor parasitism of planthopper eggs, we prepared sentinel rice plants (IR66) with different densities of *N. lugens* eggs. Rice plants were transplanted to #3 pots (9.5 × 12.5 cm, H × D) in soil collected from each type of nitrogen treatment plot (i.e., soils under three nitrogen regimes). The sentinel plants had been sown to dry seedbeds (on 9 May 2011) together with the rice plants in the paddy fields and were transplanted to the pots at the same time (6 June 2011) as transplanting in the field. The sentinel plants were covered with acetate cages of 40 × 12 cm (H × D) that fitted neatly into the pots. The cages had a mesh top and mesh windows. Gravid female *N. lugens* were taken from a colony maintained at IRRI and placed on the plants at densities of 1, 2, 3, 4 and 5 females per plant. The planthoppers were allowed to feed and lay eggs for 24 h, after which time they were removed, and the plants transported to the field. The plants were set out in each field plot as a block (i.e., with eggs from 1 to 5 females; *n* = 8) during the early morning and were collected 72 h later. Plants were returned to the laboratory and the eggs and parasitoids allowed develop for a further 10 days in large glass tubes (25 × 3 cm, H × D) with moistened filter paper. After 10 days, the tubes were examined for adult parasitoids that had emerged from the eggs, and the plants dissected to determine the number and fate of unhatched eggs.

To monitor the mortality of stemborer eggs, adult female moths were collected from rice fields in the Laguna area around IRRI. The moths were maintained in large acetate cages with 30-day-old rice plants for 24 h and allowed to oviposit on the rice plants. Egg masses were collected from the cage by cutting the leaf tissue behind the masses. These were transferred to the IR66 sub-plots at 30 and 60 DAT (on 25 July 2011 and 24 August 2011, respectively) and attached to rice plants in the plots using double-sided sticky tape. The egg masses were set-out in each field as blocks at densities of 1, 2, 3, 4, and 5 egg masses per rice hill. Egg masses were set out in the early morning and were collected 72 h later. The masses were held in individual glass vials until the adult parasitoids or stemborer larvae emerged. Egg masses were then examined for unhatched eggs.

### 2.6. Data Analyses

We examined the effects of plot management (nitrogen, varieties, bund type) and sampling periods on arthropod communities by assigning species and morphospecies to groups of interest. These groups included four types of rice pest—leafhoppers, planthoppers, lepidoptera and grain-sucking bugs. Because the rice arthropod community has been studied extensively, particularly in the Philippines [52], we identified pests to species level and assigned species as follows: Leafhopper (Hemiptera: Cicadellidae) vectors of tungro disease—*Nephotettix virescens* (Distant), *N. malayanus* Ishihara & Kawas, *N. nigropictus* (Stål) and *Recilia dorsalis* (Motschulsky); planthoppers (Hemiptera: Delphacidae)—*Nilaparvata lugens* and *Sogatela furcifera* (Horváth); lepidopteran pests of rice including Pyralidae—*Chilo suppressalis* (Walker), *Chaphalocrocis medinalis* (Guenee), *Marasmia patnalis* Bradley; Noctuidae*—Sesamia inferens* (Walker)*, Rivula atimeta* (Swinhoe), and Crambidae—*Scirpophaga innotata* (Walker) and *S. incertulas*; grain-sucking bugs—*Leptocorisa oratorius* (Fabricius) (Hemiptera: Alydidae) and grain-sucking Lygaeidae (Hemiptera). Together, these pest species accounted for 73.7% of all herbivores in the samples.

We also identified most natural enemies of rice pests to species or genus level based on several publications from the Philippines [52,54]. Natural enemies were assigned as predatory beetles (Coleoptera) including Coccinellidae—*Micraspis crocea* (Mulsant) and *Harmonia octomaculata* (Fabricius); Staphylinidae—*Paederus* spp. and *Philonthus* sp., and Carabidae—*Ophionea nigrofasciata* Schmidt-Gobel and other carabid species reported as predators in rice fields [52,54]; predatory bugs (Hemiptera) including *Cythorhinus lividipennis* Reuter (Miridae), *Anisops* sp. (Notonectidae), *Limnogonus fossarum* (Fabricius) (Gerridae), *Mesovelia vittigera* Horváth (Mesoveliidae), *Micronecta* sp. (Corixidae), *Saldula ornatula* (Reuter) (Saldidae), *Polytoxus fuscovittatus* (Stål) (Reduviidae), *Cymodema basicornis* (Motschulsky) (Cymidae) and *Nabia* spp. (Nabiidae); predatory flies (Diptera) including *Anatrichus pygmeus* Lamb (Chloropidae), *Culicoides* sp., *Nilobezzia acanthopus* (de Meijere) and *Stilobezzia longistyla* Tokunaga (Ceratopogonidae), *Drapetis (Elaphropeza)* sp. (Hybotidae), *Ochthera brevitibialis* de Meijere (Ephydridae), *Sepedon sphegea* (Fabricius)(Sciomyzidae), and other flies reported as predators in rice fields [52,54]; dragonflies and damselflies (Odonata)—*Agriocnemis* spp. (Coenagrionidae), *Crocothemis* spp., *Diplacodes* spp., and *Orthetrum* spp. (Libellulidae) and all larval forms of Odonata; spiders (Araneae) that were mainly *Tetragnatha* spp. (Tetragnathidae) and *Pardosa pseudoannulata* (Bösenberg & Strand) (Lycosidae), but including all spiders and spiderlings; the egg parasitoids (Hymenoptera) of grain-sucking bugs—*Gryon nixoni* Masner (Scelionidae) and *Psix lacunatus* Johnson & Masner (Platygastridae); parasitoids of the eggs and free-living stages of rice planthoppers and leafhoppers including wasps (Hymenoptera) such as *Anagrus* spp. and *Gonatocerus* spp. (Mymaridae), *Oligosita* spp. (Trichogrammatidae), and *Pseudogonatopus flavifemur* Esaki & Hashimoto (Dryinidae) and flies (Diptera) such as *Tomosvaryella oryzaetora* (Koizumi) and *Pipunculus javanensis* (de Meijere) (Pipunculidae) [52,55]; and parasitoids (Hymenoptera) of the eggs and larvae of lepidopteran pests—*Telenomus* spp. (Platygastridae), *Tetrastichus* spp. (Eulophidae), *Trichogramma* spp. (Trichogrammatidae), *Temelucha philippinensis* (Ashmead), *Itoplectis narangae* (Ashmead)(Ichneumonidae) and *Trichomma cnaphalocrosis* Uchida (Ichneumonidae), *Bracon chinensis* (Szepl.), *Tropobracon schoenobii* (Viereck), *Cardiochiles philippinensis* Ashmead, *Cotesia angustibasis* (Gahan), and *Macrocentrus philippinensis* Ashmead *(*Braconidae), and *Goniozus* spp. (Bethylidae), as well as other species reported as parasitoids of lepidopteran rice pests [52,56]. These natural enemy species accounted for 96.3% of predators and 72.7% of parasitoids collected during sampling. Further details of the species composition of the rice arthropod communities will be presented in a future paper.

We use repeated-measure general linear models (GLM) to examine trends in the abundance of each pest and natural enemy group during the 2011 and 2013 wet season crops. In 2011, the GLMs accounted for sampling period (repeated-measure), nitrogen level, and adjacent bund type. For 2011, we examined the fauna on the bunds separately using univariate GLM because of the different sampling techniques for rice and bund arthropods in that year. We initially included field as a blocking factor in all analyses, but removed this where there were no effects. In 2013, we used repeated-measure GLMs to examine the effects of sampling period (repeated-measure), nitrogen level, and habitat (including rice types and bund types). We also used multivariate GLM to examine leafhopper and planthopper communities and their principal predators and parasitoids during early crop stages in 2013 (based on 1 August 2013 samples). These included nitrogen level, rice variety, and habitat type as main factors and removing the effects of block. Nitrogen levels were treated as the main plots and habitat type (bund type) and variety as sub-plots during analyses. Tukey tests were used to determine homogenous nitrogen and habitat (2013) groups. We used univariate GLMs to examine the development of vegetation on the rice bunds (factors = nitrogen levels and bund type) and rice yields (factors = nitrogen levels, bund type, and variety).

In 2011 we investigated regulation of planthopper and stemborer eggs by egg parasitoids. Data was initially assessed for density dependence within field sub-plots. However, we did not find density dependence of planthopper egg parasitim (possibly due to high levels of egg predation, typically ca 45%) and only weak density dependence of stemborer egg parasitism. Therefore, in the present study we combined parasitism rates from sentinel plants within sub-plots. Parasitism was analyzed using repeated measure GLMs as total parasitism (i.e., the combined effect of all parasitoid species) and parasitism due to each of the principal parasitoid genera. Models examined the effects of sampling period (repeated-measure), nitrogen levels, and bund type on percent parasitism and included within subject and between subject interactions.

Residuals were plotted following all parametric analyses to test for normality and homogeneity. Where data were not normal and homogenous, we applied transformations and reexamined the residuals after rerunning analyses in a stepwise fashion until data met the requirements for parametric analysis. Final transformations are indicated with the results. Where data could not be normalized, we ranked cases. All statistical analyses were conducted using SPSS version 23.0 (IBM SPSS Statistics, Armonk, NY, USA). We only report significant effects from the study.

## 3. Results

### 3.1. Development of Bund Vegetation

Bund management affected the richness and biomass of vegetation on the bunds during the 2011 WS (Table 1). Weedy bunds had similar floral diversity to the cleared bunds, but because the weeds were not mowed, the bund vegetation attained a higher biomass. Okra survived better on the bunds (100% survival of plants) than sesame (75% survival). The sesame/okra bunds were lightly weeded after sowing at the beginning of the crop season, but required no mowing as the vegetable plants developed (Table 1). Production (yield) of sesame and okra was not quantified during 2011.

Mowing the cleared bunds during the 2013 WS maintained a low biomass of weeds compared to the mung bean bunds (Table 2). The height and productivity of the mung bean plants were affected by nitrogen levels in the adjacent rice plots with larger plants and higher productivity on bunds adjacent to high-nitrogen plots (Table 2).

### 3.2. Rice Yields

Nitrogen affected rice yields, with the lowest production in plots with zero added nitrogen in both years, and highest production in N3 plots during 2011 (Table 1), and in N2 and N3 plots in 2013 (Table 2). During 2013, IR62 plants attained consistently higher yields than the IR64 plants. There was no significant effect of bund vegetation on rice yields in either 2011 or 2013 (Table 1 and Table 2).

### 3.3. Arthropod Communities during the 2011 Wet Season Experiment

**Figure 3:**
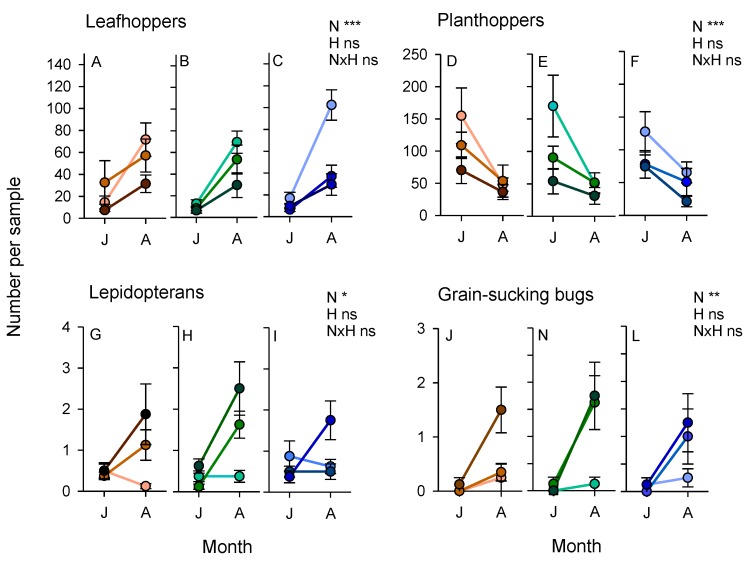
Relative abundance of principal rice herbivores from sweepnet sampling of rice plots during July (J) and August (A) 2011. The abundance of leafhopper vectors of tungro virus (**A**–**C**), planthoppers (**D**–**F**), lepidopterans including stemborers and leaffolders (**G**–**I**) and grain sucking bugs (**J**–**L**) are indicated. Plots were planted with IR66 and received one of three nitrogen-fertilizer treatments (indicated by color intensity of points, N1 = light, N2 = medium, N3 = dark). Samples were collected at < 3 m from clear bunds (brown lines: A, D, G, J), weedy bunds (green lines: B, E, H, N) or sesame/okra bunds (blue lines: C, F, I, L). Standard errors are indicated (*n* = 8). General linear models (GLM) results for effects of nitrogen (N), bund type (H) and their interactions (N × H) are indicated for each herbivore group; ns = *p* > 0.05, * = *p* < 0.05, ** = *p* < 0.01, *** = *p* < 0.001.

A total of 28,844 arthropod specimens were captured during sweepnet sampling in July and August 2011. Abundance of the main pest groups was affected by sampling period with leafhoppers (within subject effect (sampling period): F_1,63_ = 119.290, *p* < 0.001), lepidopterans (F_1,63_ = 7.636, *p* < 0.01) and grain-sucking bugs (F_1,63_ = 51.371, *p* < 0.001) increasing in abundance as the crop matured, but planthoppers (F_1,63_ = 33.800, *p* < 0.001) declining in abundance over time (Figure 3). Nitrogen affected abundance, with leafhoppers (F_2,63_ = 9.480, *p* < 0.001) and planthoppers (F_2,63_ = 9.317, *p* < 0.001) being more abundant in samples from low-nitrogen plots (Figure 3) and lepidopterans (F_2,63_ = 3.892, *p* < 0.05) and rice bugs (F_2,63_ = 8.251, *p* < 0.001) being most abundant in the high-nitrogen plots irrespective of bund treatments (Figure 3). There were significant sampling period × nitrogen interactions for leafhoppers (F_2,63_ = 5.406, *p* < 0.01) and lepidopterans (F_2,63_ = 3.195, *p* < 0.05) because of similar abundance across nitrogen treatments during the early crop stage, but marked preferences for low or high-nitrogen plots, respectively, at the later crop stage (Figure 3). There was no effect of bund type on pest abundances during the 2011 wet season (3.892 > F_2,63_ < 9.480, *p* > 0.05).

**Figure 4:**
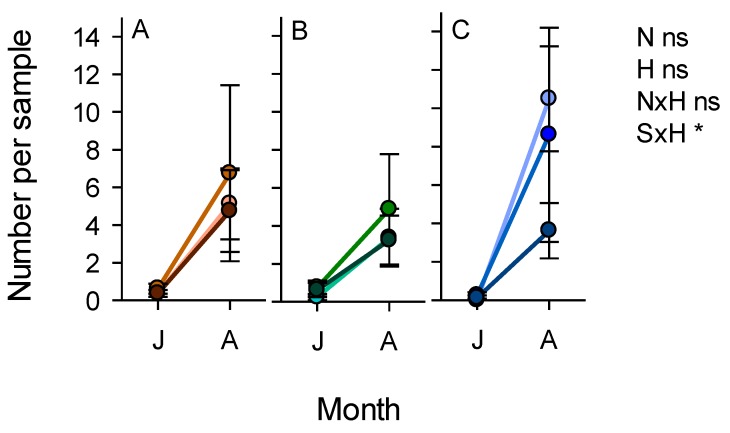
Relative abundance of predatory bugs—mainly *Cythorhinus lividipennis*—based on sweepnet samples from rice plots during July (J) and August (A) of 2011. Plots were planted with IR66 and received one of three nitrogen-fertilizer treatments (indicated by color intensity of points, N1 = light, N2 = medium, N3 = dark). Samples were collected < 3 m from clear bunds (brown line, **A**), weedy bunds (green lines, **B**) or sesame/okra bunds (blue lines, **C**). Standard errors are indicated (*n* = 8). GLM results for effects of nitrogen (N), bund type (H) and their interaction (N × H), and the interaction between crop stage and bund type (S × H) are indicated; ns = *p* > 0.05, * = *p* < 0.05. See Supplementary Figure S1 for full details of predators in the rice plots.

The abundance of predatory beetles (within subject effect (sampling period): F_1,63_ = 102.088, *p* < 0.001), predatory bugs (F_1,63_ = 62.747, *p* < 0.001), dragonflies and damselflies (F_1,63_ = 32.670, *p* < 0.001), and parasitoids of planthoppers and leafhoppers (F_1,63_ = 7.721, *p* < 0.05) increased as the crop matured, whereas the abundance of predatory flies (F_1,63_ = 49.988, *p* < 0.001) and spiders (F_1,63_ = 35.539, *p* < 0.001) declined as the crop matured. Parasitoids of lepidopteran herbivores and rice bugs were generally stable throughout crop development (Supplementary Figure S1). Beetles (F_2,63_ = 5.160, *p* < 0.01) were more abundant in high-nitrogen plots, whereas spiders (F_2,63_ = 5.008, *p* < 0.01) were more abundant in low-nitrogen plots (Supplementary Figure S1). The only apparent effect of bunds was a sampling date (rice stage) × bund interaction for predatory bugs (F_2,63_ = 4.057, *p* < 0.05) (Figure 4). This was due to higher abundances of the predatory mirid bug, *C. lividipennis*, in rice plots adjacent to sesame/okra bunds during the rice reproductive stage, but a similar low abundace of the mirid in all plots (regardless of adjacent bund type) during the early crop stage (Figure 4).

**Figure 5:**
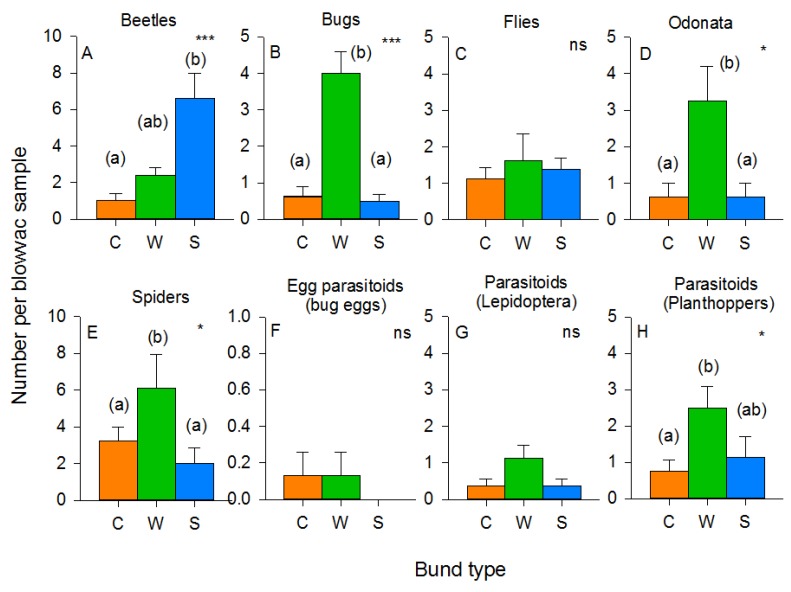
Relative abundance of the natural enemies of rice herbivores on clear bunds (C, brown bars), weedy bunds (W, green), and sesame bunds (S, blue) during sampling conducted on 1 September 2011. Graphs indicate the numbers of predatory beetles (**A**), bugs (**B**), flies (**C**), dragonflies and damselflies (**D**), and spiders (**E**) captured during Blow-vac sampling. The numbers of parasitoids of rice bugs (**F**), lepidopteran pests (**G**), and planthoppers/leafhoppers (**H**) are also indicated. GLM results for the effects of bund vegetation are indicated for each predator group; ns = *p* > 0.05, * = *p* < 0.05, *** = *p* < 0.001. Lowercase letters indicate homogenous bund groups.

A total of 3762 arthropod specimens was captured on bunds using the Blow-vac sampler. There were no effects of bund type on the abundance of leafhopper, planthopper or lepidopteran herbivores (0.055 > F_2,21_ < 2.328, *p* > 0.05) (Supplementary Figure S2), and no ricebugs were captured. The abundance of predatory beetles (F_2,21_ = 9.175, *p* < 0.001) was highest on sesame/okra bunds, whereas predatory bugs (F_2,21_ = 17.637, *p* < 0.001), dragonflies and damselflies (F_2,21_ = 3.893, *p* < 0.05), spiders (F_2,21_ = 3.301, *p* < 0.05), and planthopper parasitoids (F_2,21_ = 4.464, *p* < 0.05) were most abundant on the weedy bunds (Figure 5).

### 3.4. Arthropod Communities during the 2013 Wet Season Experiment

**Figure 6:**
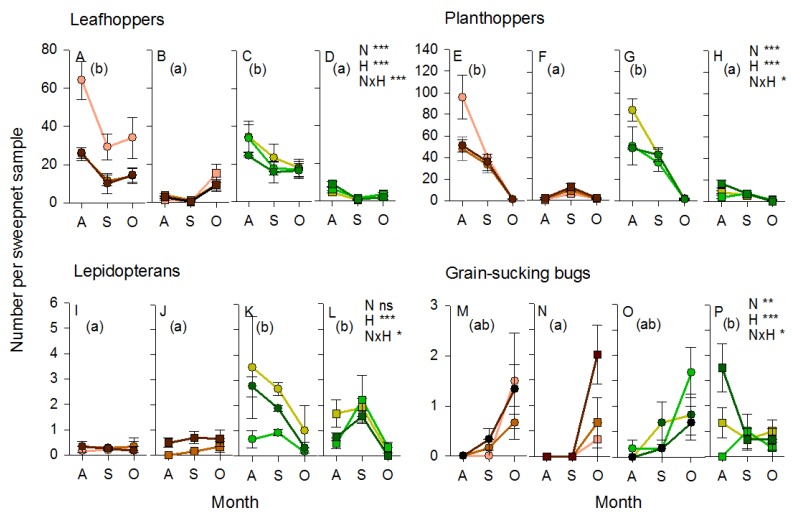
Relative abundance of principal rice herbivores from sweepnet sampling of rice plots during August (A), September (S) and October (O) of 2013. Samples from clear bunds and adjacent rice are indicated by brown lines and symbols with samples from mung bean bunds and adjacent rice indicated by green lines and symbols. The abundance of leafhopper vectors of tungro virus (**A**–**D**), planthoppers (**E**–**H**), lepidopterans including stemborers and leaffolders (**I**–**L**), and grain sucking bugs (**M**–**P**) are indicated. Plots were planted with IR64 and received one of three nitrogen-fertilizer treatments (indicated by color intensity of points, N1 = light, N2 = medium, N3 = dark). Samples were collected from the rice crop (circles, A, C, E, G, I, K, M, O) and from bund vegetation (square symbols, B, D, F, H, J, L, N, P). Samples from rice were collected at < 3 m from clear bunds (A, E, I, M), or vegetable bunds (C, G, K, O). Standard errors are indicated (*n* = 8). GLM results for the effects of nitrogen (N), habitat type (H) and their interactions (N × H) are indicated for each herbivore group; ns = *p* > 0.05, * = *p* < 0.05, ** = *p* < 0.01, *** = *p* < 0.001. Lowercase letters indicate homogenous habitat groups.

A total of 38,242 arthropods specimens was captured in the plots and on bunds using sweepnet sampling in 2013. As with the 2011 samples, the abundance of planthoppers declined (within subject effect [sampling period]: F_1,60_ = 156.188, *p* < 0.001) and rice bugs increased (F_1,60_ = 32.674, *p* < 0.001) in the IR64 rice plots as the crop matured (Figure 6). However, planthoppers were very scarce on the bunds, and rice bugs declined on mung bean bunds over the season producing significant sampling period (crop stage) × habitat type interactions (planthoppers: F_1,60_ = 5.968, *p* < 0.01; rice bug: F_1,60_ = 9.614, *p* < 0.001) (Figure 6). In contrast to 2011, the abundance of leafhoppers declined as the crop matured (F_1,60_ = 15.563, *p* < 0.001) (differences in trends between the two seasons may relate to differences between the ontogeny of resistance in IR66 and IR64), but with relatively stable numbers occurring on the bund vegetation. This produced a significant sampling period (crop stage) × habitat type interaction (F_2,60_ = 12.382, *p* < 0.001) (Figure 6). Lepidopteran herbivores were relatively stable on the clear bunds and adjacent rice, but declined from a high abundance (see below) during the early crop stages on the mung bean bunds and adjacent rice. This produced a further significant sampling period (crop stage) × habitat type interaction (F_1,60_ = 6.165, *p* < 0.001) (Figure 6).

Leafhoppers (F_1,60_ = 12.712, *p* < 0.001) and planthoppers (F_1,60_ = 5.399, *p* < 0.01) were more abundant in low-nitrogen plots. For planthoppers, this was most apparent during the early vegetative crop stages (sampling period × nitrogen: F_2,60_ = 5.968, *p* < 0.001) and in rice habitat (nitrogen × habitat: F_6,60_ = 2.522, *p* < 0.05) (Figure 6). Lepidopteran herbivores were more abundant on clear bunds adjacent to high-nitrogen plots, and in low-nitrogen mung bean bunds and adjacent rice plots (nitrogen × habitat: F_6,60_ = 3.048, *p* < 0.01) with differences most apparent during the early crop stages (sampling period × habitat: F_3,60_ = 12.382, *p* < 0.001) (Figure 6). Leafhoppers (F_3,60_ = 64.802, *p* < 0.001) and planthoppers (F_3,60_ = 70.345, *p* < 0.001) had consistently low abundances on bunds compared to adjacent rice plots. In contrast, lepidopteran herbivores (F_1,60_ = 19.004, *p* < 0.001) were most abundant on mung bean bunds and adjacent rice, and ricebug was most abundant on rice adjacent to mung bean bunds (F_1,60_ = 3.328, *p* < 0.05) (Figure 6).

**Figure 7:**
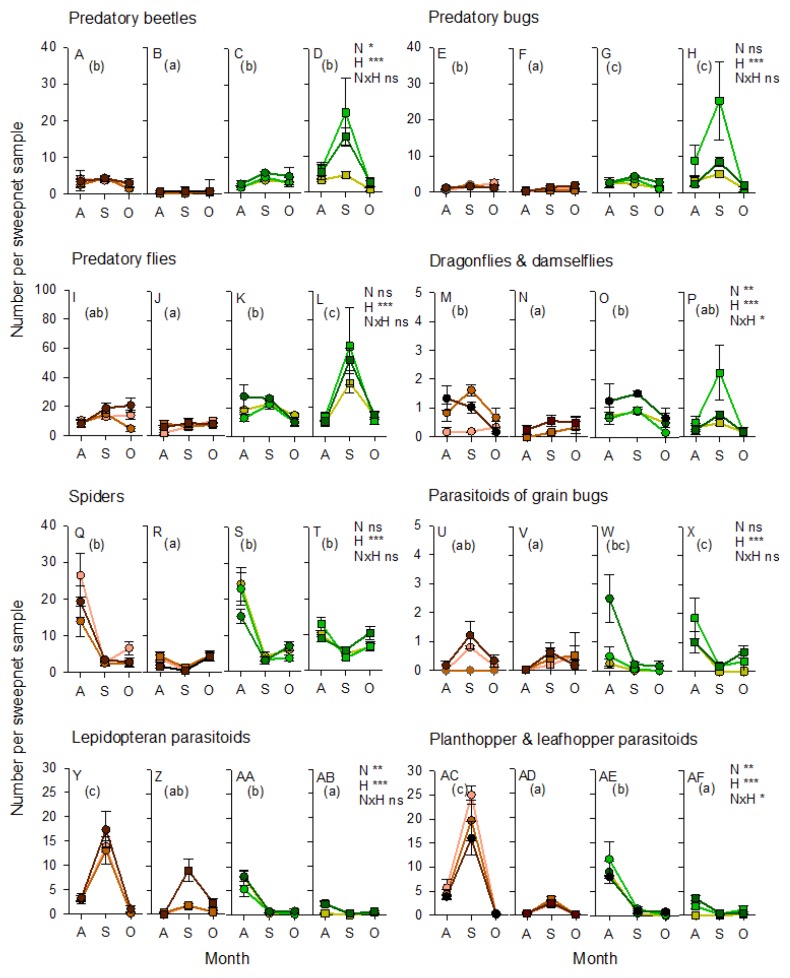
Relative abundance of the principal natural enemies of rice herbivores from sweepnet sampling of rice plots during August (A), September (S), and October (O) of 2013. Samples from clear bunds and adjacent rice are indicated by brown lines and symbols with samples from mung bean bunds and adjacent rice indicated by green lines and symbols. The abundance of predatory beetles (**A**–**D**), predatory bugs (**E**–**H**), predatory flies (**I**–**L**), dragonflies and damselflies (**M**–**P**), spiders (**Q**–**T**), egg parasitoids of grain bugs (**U**–**X**), larval and egg parasitoids of lepidopteran herbivores (**Y**–**AB**), and egg and nymph parasitoids of planthoppers and leafhoppers (**AC**–**AF**) are indicated. Plots were planted with IR64 and received one of three nitrogen-fertilizer treatments (indicated by color intensity of points, N1 = light, N2 = medium, N3 = dark). Samples were collected from the rice crop (circles, A, C, E, G, I, K, M, O, Q, S, U, W, Y, AA, AC, AE) and from bund vegetation (square symbols, B, D, F, H, J, L, N, P, R, T, V, X, Z, AB, AD, AF). Standard errors are indicated (*n* = 8). GLM results for the effects of nitrogen (N), habitat type (H) and their interactions (N × H) are indicated for each predator/parasitoid group; ns = *p* > 0.05, * = *p* < 0.05, ** = *p* < 0.01, *** = *p* < 0.001. Lowercase letters indicate homogenous habitat groups.

Predatory beetles, mainly *M. crocea*, were abundant in both the rice and mung bean habitat, but were largely absent from the cleared bunds (F_3,60_ = 27.339, *p* < 0.001). Beetles were also more abundant in the high-nitrogen habitats (F_2,60_ = 4.235, *p* < 0.05) (Figure 7). Predatory bugs, mainly *C. lividipennis* (F_3,60_ = 32.489, *p* < 0.001), predatory flies (F_3,60_ = 18.927, *p* < 0.001), dragonflies and damselflies (F_3,60_ = 7.890, *p* < 0.001), and the parasitoids of rice bugs, particularly *Gryon nixoni* (F_3,60_ = 7.168, *p* < 0.001), were most abundant in the mung bean habitat and adjacent rice (Figure 7). The parasitoids of lepidopteran herbivores (F_3,60_ = 24.593, *p* < 0.001) and planthoppers and/or leafhoppers (F_3,60_ = 33.643, *p* < 0.001) were more abundant in the rice habitat, particularly in rice adjacent to the clear bunds. Parasitoids of lepidopteran eggs and caterpillars (F_2,60_ = 6.905, *p* < 0.01), predatory beetles (F_2,60_ = 4.235, *p* < 0.05), as well as dragonflies and damselflies (F_2,60_ = 5.211, *p* < 0.01), were more abundant in the high-nitrogen habitats (albeit with a significant nitrogen × habitat interaction, F_2,60_ = 2.678, *p* < 0.05, for dragonflies because of a high abundance of low-nitrogen mung beans), whereas the parasitoids of planthoppers were more abundant in low-nitrogen habitats (F_2,60_ = 6.476, *p* < 0.01), particularly the low-nitrogen rice plots (nitrogen × habitat: F_6,60_ = 2.539, *p* < 0.05) (Figure 7). Spiders (F_1,60_ = 80.646, *p* < 0.001) and hymenopteran parasitoids of rice bugs (F_2,60_ = 34.428, *p* < 0.001); lepidopterans (F_2,60_ = 57.712, *p* < 0.001) and planthoppers (F_2,60_ = 209.064, *p* < 0.001) were most abundant in the early crop stages, particularly in the rice habitat (sampling period × habitat: parasitoids of lepidopterans F_6,60_ = 24.593, *p* < 0.001; parasitoids of planthoppers F_6,60_ = 20.677, *p* < 0.001) (Figure 7).

### 3.5. Parasitism of Planthopper and Stemborer Eggs (2011 Experiment)

**Figure 8:**
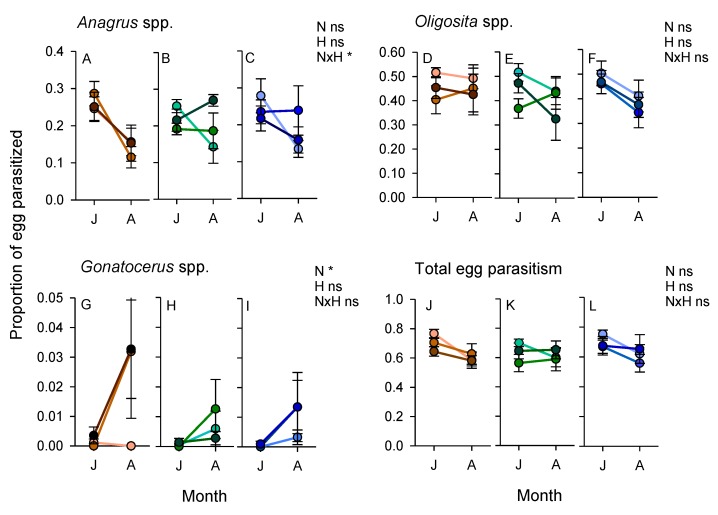
Parasitism of planthopper eggs in rice plants adjacent to clear bunds (brown lines: A, D, G, J), weedy bunds (green lines: B, E, H, K), and sesame bunds (blue lines: C, F, I, L) during July (J) and August (A) of the 2011 wet season crop. Graphs indicate parasitism by *Anagrus* spp. (**A**–**C**), *Oligosita* spp. (**D**–**F**), and *Gonatocerus* spp. (**G**–**I**) as well as the total parasitism (**J**–**L**). Plots were planted with IR66 and received one of three nitrogen-fertilizer treatments (indicated by color intensity of points, N1 = light, N2 = medium, N3 = dark). Standard errors are indicated (*n* = 8). GLM results for the effects of nitrogen (N), habitat type (H) and their interactions (N × H) are indicated for each parasitoid group; ns = *p* > 0.05, * = *p* < 0.05. Lowercase letters indicate homogenous habitat groups.

Planthoppers laid 20–24% fewer eggs on potted IR66 plants that received added nitrogen (i.e., N2 and N3: F_2,66_ = 6.214, *p* < 0.01) and laid 25% fewer eggs on older rice plants (plant stage: F_1,66_ = 19.585, *p* < 0.001). The mortality of planthopper eggs exposed in the rice fields during 2011 was high. Between 70 and 86% of all eggs were killed by parasitism from a range of egg parasitoids and from predation by unidentified predators during 3 days of exposure. *Oligosita* spp. were the main parasitoids, impacting over 50% of eggs (Figure 8). Parasitism was generally highest during the early crop stages (within subject effect (sampling period)—total parasitism: F_1,60_ = 7.124, *p* < 0.01; *Anagrus* spp.: F_1,60_ = 15.824, *p* < 0.001, *Oligosita* spp.: F_1,60_ = 6.196, *p* < 0.01); however, parasitism by *Gonatocerus* spp. was highest at later crop stages (F_1,60_ = 10.140, *p* < 0.01). Parasitism was largely unaffected by the vegetation on adjacent bunds (0.050 < F_2,60_ < 1.884, *p* > 0.05) and only parasitism by *Gonatocerus* spp. was affected by nitrogen levels, with higher rates of parasitism in high-nitrogen plots (F_2,60_ = 3.008, *p* < 0.05) (Figure 8). There was a significant nitrogen × bund type interaction for *Anagrus* spp. parasitism because of higher parasitism of eggs in high-nitrogen plots (N2 and N3) adjacent to weedy and sesame/okra bunds compared to similar plots adjacent to clear bunds (F_4,60_ = 2.09, *p* < 0.5) (Figure 8).

**Figure 9:**
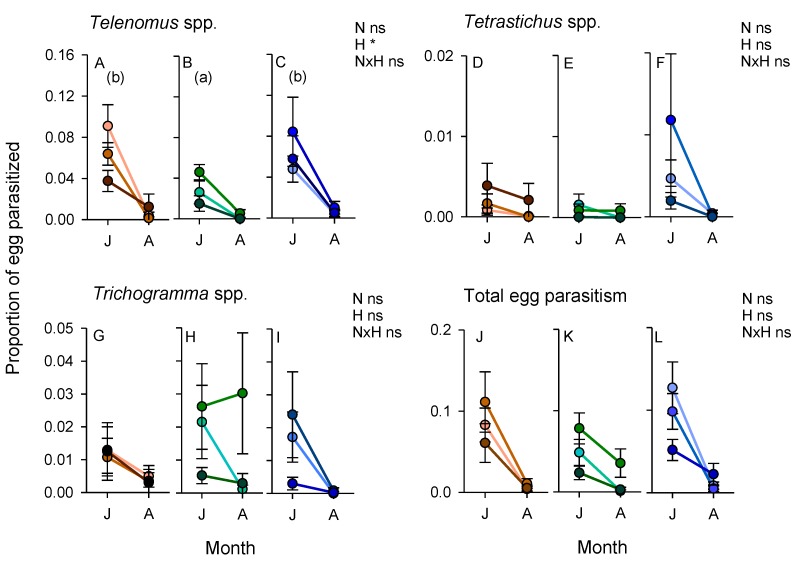
Parasitism of stemborer eggs on rice plants adjacent to clear bunds (brown lines: A, D, G, J), weedy bunds (green lines: B, E, H, K), and sesame bunds (blue lines: C, F, I, L) during July (J) and August (A) of the 2011 wet season crop. Graphs indicate parasitism by *Telenomus* spp. (**A**–**C**), *Tetrastichus* spp. (**D**–**F**), and *Trichogramma* spp. (**G**–**I**) as well as the total parasitism (**J**–**L**). Plots were planted with IR66 and received one of three nitrogen-fertilizer treatments (indicated by color intensity of points, N1 = light, N2 = medium, N3 = dark). Standard errors are indicated (*n* = 8). GLM results for the effects of nitrogen (N), habitat type (H) and their interactions (N × H) are indicated for each parasitoid group; ns = *p* > 0.05, * = *p* < 0.05. Lowercase letters indicate homogenous habitat groups.

Parasitism of stemborer eggs was <20% during field exposures. Parasitism by all species was higher during the early crop stage (within subject effects (sampling period)—*Telenomus*: F_1,60_ = 164.414, *p* < 0.001; *Tetrastrichus*: F_1,60_ = 8.798, *p* < 0.01; *Trichogramma*: F_1,60_ = 24.170, *p* < 0.001; total parasitism: F_1,60_ = 8.798, *p* < 0.01) (Figure 9). Parasitism was not affected by nitrogen levels (0.068 < F_2,60_ < 2.853, *p* > 0.05) (Figure 9). *Telenomus* spp. were the most common parasitoids encountered during the experiment. Parasitism by *Telenomus* spp. was significantly lower in rice adjacent to weedy bunds than in rice adjacent to other bund types (F_2,60_ = 4.250, *p* < 0.05) (Figure 9).

### 3.6. Combined Effects of Nitrogen, Variety and Bund Vegetation (2013 Experiment)

**Figure 10:**
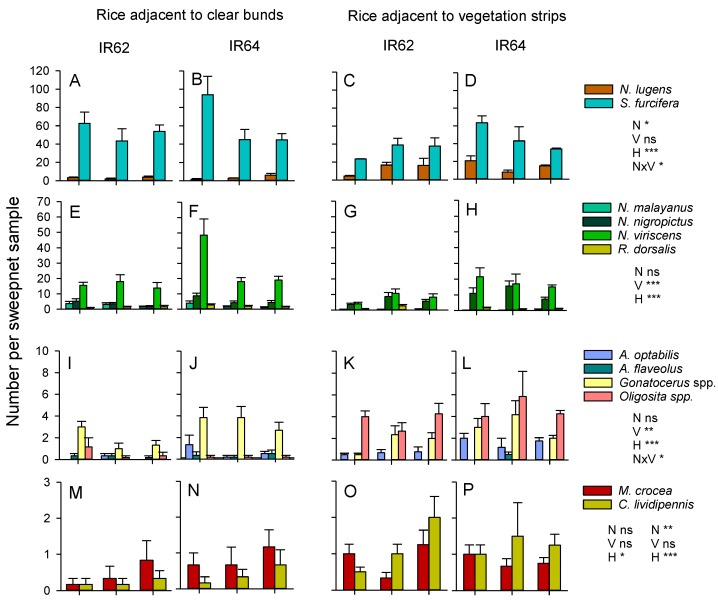
Relative abundance of planthoppers, leafhoppers and their specialist natural enemies in rice plots adjacent to clear bunds (A,B,E,F,I,J,M,N) and mung bean bunds (C,D,G,H,K,L,O,P) during the early crop stage (August) of the 2013 wet season. Plots were treated with one of three levels of nitrogen (N1, N2, N3) and planted with IR62 (resistant, A,C,E,G,I,K,M,O) or IR64 (susceptible, B,D,F,H,J,L,N,P) rice varieties. Graphs indicate the abundance of planthoppers (*N. lugens* and *S. furcifera*) (**A**–**D**), leafhopper vectors of tungro virus (*N. malayanus*, *N. negropictus*, *N. virescens* and *R. dorsalis*) (**E**–**H**), egg parasitoids (*A. optabilis*, *A. flaveolus*, *Gonatocerus* spp. and *Oligosita* spp.) (**I**–**L**), and two specialist predators (*M. crocea* and *C. lividipennis*) (**N**–**P**). Standard errors are indicated (*n* = 6). Multiple GLM results for the effects of nitrogen (N), variety (V), bund type (H) and their significant interactions (N × V) on all species are presented in the text. Between group effects are indicated below the legends for each species group, except predators where results for *M. crocea* (on left) and *C. lividipennis* (on right) are presented separately. ns = *p* > 0.05, * = *p* < 0.05, ** = *p* = 0.01, *** = *p* < 0.005.

We only considered the effects of varieties on planthoppers, leafhoppers and their specialist or principal natural enemies. This was because both IR62 and IR64 contain currently effective (IR62) and non-effective (IR64) planthopper resistance genes. Our results showed no significant effect of variety on the composition of the planthopper communities (F_2,59_ = 1.457, *p* > 0.05) or the abundance of either of the two species, *N. lugens* (F_1,60_ = 0.507, *p* > 0.05) or *S. furcifera* (F_1,60_ = 2.896, *p* > 0.05) (Figure 10). Variety affected leafhopper communities (F_4,57_ = 8.396, *p* < 0.001), with lower abundances of *N. nigropictus* (F_1,60_ = 9.463, *p* < 0.01) and *N. virescens* (F_1,60_ = 23.384, *p* < 0.001) on the resistant variety. Nitrogen levels affected the planthopper community (F_4,120_ = 131.544, *p* < 0.05), but had no apparent effect on the leafhopper community (F_8,114_ = 1.064, *p* > 0.05). For planthoppers, this was mainly due to a decline in the abundance of *S. furcifera* in high-nitrogen plots (F_2,60_ = 3.753, *p* < 0.05) (Figure 10). There were also significant nitrogen × variety interactions for *S. furcifera* (F_2,60_ = 4.121, *p* < 0.05), *N. virescens* (F_2,60_ = 4.766, *p* < 0.01), and *R. dorsalis* (F_2,60_ = 4.112, *p* < 0.05), because of generally se and lower abundances on IR62 across nitrogen levels but declining abundance on IR64 as nitrogen levels increased (Figure 10). Habitat (bund vegetation) had the greatest effects on the planthopper (F_2,59_ = 24.912, *p* < 0.001) and leafhopper (F_4,57_ = 12.718, *p* < 0.001) communities. *S. furcifera* had a lower abundance in plots adjacent to mung bean bunds (F_1,60_ = 7.356, *p* < 0.01), whereas *N. lugens* (F_1,60_ = 31.274, *p* < 0.001) had higher abundance in the same plots (Figure 10). *N. malayanus* (F_1,60_ = 25.939, *p* < 0.001) and *N. virescens* (F_1,60_ = 19.662, *p* < 0.001) were all less abundant in plots adjacent to mung bean, compared to plots adjacent to cleared bunds, but *N. nigropictus* was more abundant close to the mung bean bunds (F_1,60_ = 7.776, *p* < 0.01) (Figure 10).

Variety (F_4,57_ = 4.354, *p* < 0.01) and bund vegetation (F_4,57_ = 31.520, *p* < 0.001) affected the community of egg parasitoids in the rice plots, but nitrogen levels had no apparent effect (F_8,114_ = 0.875, *p* > 0.05) (Figure 10). *A. optabilis* (F_1,60_ = 9.522, *p* < 0.01) and *Gonatocerus* spp. (F_1,60_ = 10.981, *p* < 0.01) were more abundant in plots of IR64 compared to plots of IR62 reflecting trends in the abundance of their planthopper and leafhopper hosts. *A. optabilis* (F_1,60_ = 21.322, *p* < 0.001) and *Oligosita* spp. (F_1,60_ = 102.258, *p* < 0.001) were more abundant in plots adjacent to mung bean, independently of the abundance of their hosts—which were often less abundant in rice adjacent to the mung bean plants. *A. flaveolus* (F_1,60_ = 3.915, *p* < 0.05) was more abundant in plots adjacent to cleared bunds, albeit with considerably lower abundances than the other three parasitoid species (Figure 10). There was a significant nitrogen × variety interaction effect on the abundance of *A. optabilis* (F_2,60_ = 4.651, *p* < 0.01) because of similar abundances of this species on both varieties at low nitrogen levels (N1), but higher abundances on IR64 at higher levels (N2, N3) (Figure 10). Drynid parasitoids of planthopper nymphs were also present in the samples (not indicated in Figure 10). These were more abundant in high-nitrogen plots (N2, N3) (F_2,60_ = 3.897, *p* < 0.01) of IR62 (F_1,60_ = 14.440, *p* < 0.001), adjacent to the mung bean bunds (F_1,60_ = 14.440, *p* < 0.001). The predatory coccinelid beetle, *M. crocea*, was more abundant in plots adjacent to mung bean (F_1,60_ = 5.015, *p* < 0.05), with no apparent effect of nitrogen level or variety. The mirid predator, *C. lividipennis*, was more abundant in high-nitrogen plots (F_2,60_ = 4.986, *p* < 0.05) and adjacent to mung bean bunds (F_1,60_ = 16.691, *p* < 0.001) (Figure 10).

## 4. Discussion

The benefits of using vegetation strips in rice production have been highlighted in several recent studies [14,15,16,17,21,57]. Furthermore, ecological mechanisms underlying some of these benefits, particularly as they relate to the natural regulation of rice pests, have been elucidated through laboratory and greenhouse studies [41,42,58]. Reports of potential negative effects on crop production, on farm management, or farmer productivity have been less forthcoming [19,21]. Considering the diversity of climates, topographies and socioeconomic environments under which rice is produced [59], as well as the diversity of flora and fauna present in rice fields [50,52,59], it is important to build further case-specific knowledge of the different approaches to biodiversity management and their associated positive and negative impacts. Our study indicates that there are multiple benefits to establishing floral strips on rice bunds, but that these strips require optimization to better avoid potential negative impacts. Our results over two rice cropping seasons, using three different types of vegetation strip demonstrated clear increases in the abundance of natural enemies of key rice pests compared to cleared/mowed bunds (the usual practice among rice farmers). In several cases, key predators and parasitoids were also more abundant in rice adjacent to vegetation strips; however, increased natural enemy abundance in vegetation strips was not always carried over to the adjacent rice crop. Fertilizer levels had pronounced effects on the abundance of rice pests. In a few cases (see below), vegetation strips interacted with nitrogen levels, potentially slowing the nitrogen-enhanced population growth rates of pest species. However, vegetation strips were also associated with increases in the abundance of lepidopteran and grain-sucking pests, indicating that improved designs for vegetation strips will be required to optimize benefits. The use of resistant rice varieties in combination with vegetation strips can enhance the positive impacts of either intervention. For example, we found that resistant rice adjacent to vegetation strips had a lower abundance of green leafhoppers compared to resistant rice adjacent to cleared bunds. This may have been related to higher numbers of specialist predators and parasitoids adjacent to the vegetation strips. However, a higher diversity of pests and their natural enemies close to vegetation strips also suggests that interactions within guilds are likely to be more complex and factors such as interspecific competition or indirect plant-mediated interference could have significant effects on the outcome of crop and bund management. The clear effects of bund vegetation on the diversity and abundance of the natural enemies of planthoppers during our 2013 experiment highlight the potential for vegetation strips to increase ecosystem resilience against pest outbreaks.

### 4.1. Issues of Scale

Our study was conducted over a relatively small field-plot scale. This design increased the amount of information gathered on interactions at the interface of the rice crop and vegetation strips despite practical limitations (costs and space) on our experimental design. However, to be effective, vegetation strips should have impacts in large fields of rice (typically 1 to 2 ha in Asia [49]). Furthermore, the adoption of vegetation strips has been proposed as a landscape approach to crop protection that is best practiced at community and landscape scales [14,20]. Nevertheless, several studies have indicated that poor management of rice crops can have impacts on crop health that are restricted to individual fields or farms [8,23,31,33,34]. For example, researchers interested in rice pest resurgence have observed outbreaks of planthoppers in relatively small rice plots (<10 m^2^) following the use of broad-spectrum insecticides [31,60]. Our experiments were designed to detect the effects of vegetation bunds by focusing sampling close to the bunds (<3 m, but >1 m). We assumed that the effects of the vegetation strips diminished at greater distances from the bunds [14,19]. Our experiments were designed with the intention to evaluate bund types, cognizant of the potential for bund vegetation to either have no effect or have negative effects. Without positive effects at small scales, such interventions would be inadvisable at larger scales. Despite the small plot size, our design was robust because of relatively high numbers of replicates and a high degree of interspersion between plot and bund types. Where clear trends emerged, we suggest that biologically relevant phenomena played a significant role. However, identification of these phenomena, which include vegetation strips as possible sources of food and refuges, requires further investigation.

### 4.2. Fertilizers

Fertilizers are required to meet the yield potential of modern rice varieties [25,26,59]. In our experiments, higher yields were achieved in plots with higher inputs of nitrogenous fertilizers. However, during the 2013 wet season crop, increasing nitrogen inputs from 60 to 150 Kg ha^−1^ did not result in significantly higher rice yields, indicating a greater loss of nutrient from the system at higher input levels. Much of this loss is from nutrients leaching from the rice plots [61]. During the 2013 rice crop, some of this excess nitrogen was apparently taken up by bund vegetation, resulting in larger vegetation strips and higher yields of mung bean on bunds adjacent to high-nitrogen plots. During 2011, weedy bunds adjacent to high-nitrogen plots also had the greatest vegetation biomass. During the same season, our sesame and okra plants were not affected by the nitrogen applications in the adjacent rice fields. Poor development of the sesame/okra bunds during 2011 may have been due to late planting on the bunds and competition for light with the relatively tall adjacent rice plants (as can be seen in Figure 1). To gain the best advantage from planting bunds with vegetable crops, we suggest that plants should be grown in advance of rice transplanting, such that the bund vegetation initiates flowering during the early vegetative stages of the rice crop when pest insects, such as planthoppers and leafhoppers, are most abundant [5,18]. In previous studies, a range of flowering plants and fruit or vegetable crops have been successfully produced as strips on rice bunds as part of ecological engineering. These include ornamental plants [14,17,57]; seed, bean and grain-producing flowering plants such as sesame, string beans, and mung bean [14,20,21]; fruiting plants such as bitter gourd and okra [18]; and forbs [16].

Our results support previous evidence that leaffolders and stemborers attain higher densities in high-nitrogen rice fields [23,26,32]. We also recorded a higher abundance of ricebug, *L. oratorius*, in high-nitrogen plots. However, our results differ from previous studies of the effects of nitrogenous fertilizers on planthoppers and leafhoppers [25,26,34,44], including previous studies published by our research group using field cages in the same rice plots [44]. High nitrogen has been associated with increased egg-laying, higher feeding rates, faster growth and higher survival of rice planthoppers and leafhoppers in laboratory studies [9]. Furthermore, planthoppers were more abundant on IR62 and the susceptible variety IR22 in field cages at our experimental sites during the 2011 wet season and 2012 dry season (based on an embedded experiment reported in a previous paper [44]). We suggest three possible explanations for the differences between the results we report here and previous results: Firstly, during 2011, we planted the resistant variety IR66. This variety was selected because of noted resistance against planthoppers [40]. However, the variety has received little research attention and its specific resistance mechanisms are unknown. We noted that planthoppers laid fewer eggs on IR66 plants under high nitrogen, which may suggest that resistance against planthoppers in this variety is enhanced under high nitrogen. Secondly, sweepnets capture arthropods from the tops of rice plants, whereas planthoppers and leafhoppers often feed closer to the base of the plants [21,48,49,50]. To test this idea, we made direct counts of planthoppers and leafhoppers in the rice plots during the 2011 wet season. Our counts corroborated results from the sweepnet samples, indicating that the trends were not an artefact of sampling with sweepnets. Finally, planthopper outbreaks have been associated with high fertilizer inputs, together with an overuse or misuse of insecticides [13,22,33,34,62]. Field cage experiments conducted during 2011 and 2012 in the same nitrogen-treatment plots [44] demonstrated a higher abundance of planthoppers and leafhoppers in high-nitrogen plots in the absence of natural enemies (which were excluded from the cages)(see also the results of Kenmore et al. [10]). In the present study, predatory beetles such as *M. crocea*, predatory mirid bugs such as *C. lividipennis*, predatory flies, dragonflies and damselflies, as well as the parasitoids of ricebugs and lepidopteran pests were all more abundant in high-nitrogen rice plots. Spiders (during 2011 and 2013), and the free-living stages of planthopper parasitoids (during 2013) were more abundant in low-nitrogen plots. We suggest that adult planthoppers and leafhoppers in particular, may have had higher mortality in high-nitrogen plots than in adjacent low-nitrogen plots, resulting in lower pest densities in the high-nitrogen plots at the time we conducted sampling. Higher predator abundance in high-nitrogen plots has also been reported from previous studies [23,33].

That mortality was high in our study can be seen from the very low survival of planthopper eggs (<30%) that were exposed for only three days during 2011 in the field plots. Parasitism of planthopper eggs at the experimental sites was estimated by Peñalver Cruz [63] during the 2013 wet season at about 30% using a similar methodology, with higher levels of parasitism in high-nitrogen plots. Overall, our results suggest that by maintaining a high abundance and diverse assemblage of natural enemies, the effects of high nitrogen in increasing planthopper and leafhopper fitness can be largely overcome. Furthermore, high nitrogen, although it increases host plant susceptibility [9,33,64,65], also increases plant tolerance to certain insect pests (e.g., the planthopper *S. furcifera*, and stemborers), such that rice plants better compensate for insect damage [26,44,65]. For example, although there were higher incidences of lepidopteran pests and ricebug in rice under high nitrogen, yields were apparently not affected. Rice can compensate for stemborer damage by compensatory tillering [65,66], and under certain conditions will overcompensate for herbivore damage [26,67].

### 4.3. Fertilizers and Vegetation Bunds

To be most effective, vegetation bunds should provide a stable density of natural enemies for the adjacent grain crop. They should also repel pests, or draw them away from the most vulnerable crop stages or patches of vulnerable plants (such as plants grown under high nitrogen) [14,20,68]. Our results indicate that lepidopteran and grain-sucking pests were most abundant in high-nitrogen rice plots. The presence of mung beans further increased the abundance of lepidopteran pests in the rice during 2013. Though not significant, there were also higher densities of lepidopterans in rice adjacent to weedy bunds in 2011. Similar results were noted by Vu and colleagues [19] where vegetation patches were planted to rice fields. It is unknown why lepidopterans are attracted to vegetation on the bunds; however the vegetation may provide shelter for adult moths in the otherwise open habitat of the rice fields [19,69]. Mung bean also provided a suitable habitat for ricebugs during early rice crop stages when the adjacent rice had not yet matured. These rice bugs apparently provided a food source for parasitoids such as *G. nixoni* that could then build up numbers on the bunds and in the adjacent rice. This resulted in similar densities of ricebug in rice adjacent to cleared and mung bean bunds at the time of grain filling, but higher densities of *G. nixoni* in rice adjacent to the mung bean. This improved the *G. nixoni*:ricebug ratios in the rice, and may be a mechanism to enhance the stability of ricebug populations in rice. Nevertheless, farmers who observe ricebugs on their bunds during the early season are unlikely to appreciate such potential benefits. We therefore suggest that the design of vegetation bunds should be improved to avoid the lepidopteran and grain-sucking pests of rice. This can be achieved by planting repellent or trap plants on the bunds. For example, Vetiver (*Vetiveria zizanioides*) and Napier (*Pennisetum purpureum*) grasses have demonstrated potential to attract a range of pest stemborers that subsequently fail to develop on these plants [20,70,71]. Ricebugs may also be better controlled using pheromone [72,73] or other baited traps where the bugs are concentrated along rice bunds.

During 2011, the presence of sesame and okra plants on the bunds resulted in higher abundances of mirid bugs—particularly *C. lividipennis*—in low-nitrogen plots where the free-living stages of planthoppers and leafhoppers were most abundant. *C. lividipennis* was also more abundant in rice plots adjacent to mung bean bunds in 2013. The same trends were noted with predatory flies during 2013. Previous studies have confirmed that nectar producing plants, particularly sesame, can promote the predation by *C. lividipennis* of planthoppers by increasing the longevity of the predator and improving its searching efficiency [58]. Total parasitism of planthopper or lepidopteran eggs was not affected by vegetation strips in 2011; however, the parasitism of planthopper eggs by *Anagrus* spp. was maintained at higher levels over successive sampling periods in high-nitrogen rice plots that were adjacent to weedy and sesame/okra bunds. Laboratory studies have also confirmed that *Anagrus* parasitoids are attracted to sesame, and have greater longevity and inflict higher mortality of planthopper eggs in the presence of sesame flowers [41]. In contrast to *Anagrus* spp., parasitism of stemborer eggs by *Telenomus* spp. (mainly *T. rowani*) was significantly lower in rice adjacent to weedy bunds compared to rice adjacent to either cleared or sesame/okra bunds during our 2011 experiment. This may have been due to competition with *Trichogramma* spp. wasps and the effects of contemporaneous parasitism. Using a similar method, Vu and colleagues [19] found that parasitism by *Trichogramma* spp. was significantly higher in ecologically engineered rice fields compared to control fields, suggesting that the parasitoids benefitted from food or refuges provided by the bund vegetation. This suggests that vegetation strips could be used to support biological control programs that employ *Trichogramma* spp. such as that recently described for the Mekong Region [74]. Nevertheless, the mechanisms underlying higher parasitism by *Trichogramma* spp. adjacent to vegetation strips are still unclear. This lack of knowledge may hinder precision in ecological engineering interventions of this type and indicates the need for further research attention.

Overall, our results confirm the potential for vegetation strips to enhance predation by generalist predators and parasitism. Mung beans had a greater effect on natural enemies at the beginning of the cropping seasons, but did not enhance the abundance of free-living parasitoids in adjacent rice throughout the full crop cycle. During 2013, specialist parasitoids of lepidopterans and planthoppers were most abundant in rice adjacent to cleared bunds, particularly during the mid-crop sampling in August. It is noteworthy, however, that specialist parasitoids were more abundant in rice adjacent to mung bean bunds during the early crop season, when lepidopteran and planthopper pests had their greatest densities in the rice fields and when egg-laying by planthoppers is at its peak [64]. We did not compare parasitism rates in 2013, but note that parasitism was not correlated with the abundance of free-living parasitoids during our 2011 experiments (i.e., comparing Figure 8 and Figure 9, with Supplementary Figure S1).

### 4.4. Combining Host Plant Resistance with Vegetation Strips

Nitrogen has been shown to compromise host plant resistance against rice planthoppers and leafhoppers [9,44,64]. Host resistance has also been shown to maintain plant tolerance (the ability to compensate for damage) to the brown planthopper under high nitrogen [44]. We used IR62 as a resistant host in our 2013 experiment because of its proven resistance to *N. lugens* [43,44]. Our control variety, IR64, is susceptible to planthoppers, but does have loci associated with tolerance to herbivory [45]. Our results indicated that IR62 reduced densities of *S. furcifera* in low-nitrogen rice plots compared to IR64 under similar conditions, but the effects were not apparent at higher nitrogen levels. Similar results were noted with *N. virescens*. Furthermore, both species were lower in rice plots (with both IR62 and IR64) adjacent to vegetation bunds, suggesting an additive effect. Reduced densities of both these species close to mung bean bunds were compensated for by the occurrence of other species; *N. lugens* in the case of *S. furcifera*, and *N. nigropictus* in the case of *N. virescens*. In effect, pest diversity was higher close to the vegetation bunds and the combined abundance of pests remained largely unchanged compared to rice adjacent to cleared bunds. Although a seemingly minor result, such shifts in pest communities could have important consequences for pest regulation. For example, competition between different herbivore biotypes or between herbivore species can reduce dominance by the most damaging pests thereby reducing the spread of plant viruses or preventing outbreaks [75,76]. Furthermore, interspecific competition can interact with host plant resistance to determine the structure of pest communities by further reducing resistance-targeted species [77].

In our experiments, host resistance (IR62) was associated with lower densities of egg parasitoids, but higher densities of parasitic drynid wasps. Lower densities of egg parasitoids may relate to the lower abundances of planthoppers on resistant rice, and a consequent reduction in host finding cues [9,78,79]. It may also relate to the quality of host honeydew excretions (on which parasitoids can feed). Honeydew produced by planthoppers on resistant rice is more diluted than honeydew produced from susceptible rice varieties [44]. For example, in a manipulative experiment, parasitism by *Anagrus* sp. of planthopper eggs in IR62 was enhanced by adding supplementary honeydew [63]. Therefore, alternative food sources provided by nectar-producing flowers on planted bunds [41] might also increase the effectiveness of planthopper regulation by parasitoids in plots of resistant rice. This would also inhibit the development of focal populations of adapted planthoppers (virulent against resistance), which is a considerable problem for rice breeders interested in deploying resistant varieties [8,9]. Our results indicated considerably higher numbers of parasitoids in both the resistant and susceptible rice that was adjacent to vegetation bunds, suggesting that vegetation bunds could potentially reduce adaptation rates. Furthermore, the composition of the parasitoid community was greatly influenced by proximity to mung bean bunds. Vu et al. [18] found that parasitism of rice planthopper eggs was higher close to vegetation patches, but diminished at increasing distances from the patches. In the present study, *Anagrus optabilis* and *Oligosita* spp. were more abundant in rice close to bunds with vegetation strips compared to mowed bunds, confirming the observations of Vu and colleagues. Together with the predators *C. lividipennis* and *M. crocea*, this represented a tremendous increase in the ratio of natural enemies to planthopper and leafhopper pests and confirmed that the bunds created more favourable conditions for pest regulation.

### 4.5. Further Advantages of Vegetation Strips

Our results indicate several advantages of vegetation strips. The vegetation strips enhanced the diversity of arthropods in the rice. They also provide habitat for other important arthropod groups such as pollinators [58]. The strips provided extra food and income from the field plots by utilizing parts of the field that are generally not planted with rice. Vegetation strips also utilized some of the excess fertilizer, particularly in the high-nitrogen plots, converting this to higher mung bean yields. Our experiments indicated that planned vegetation is superior to simply allowing weeds to develop. However, it should also be noted that weedy bunds had no negative impact on the rice arthropod communities; indeed, the bunds supported high numbers of beneficial insects and, despite the biomass of weeds on the bunds, there were no significant increases in pest abundance either on the bunds (compared to mowed bunds) or in the adjacent rice. Certainly, as also noted in other studies [80], the use of herbicides by farmers to clear weeds from non-crop areas could not be justified from our results. Planting vegetable crops can reduce the biomass and richness of weed communities, as occurred on our sesame/okra bunds, and allows farmers simpler management of bunds. Vegetation bunds can be associated with pests, particularly lepidopterans, and future studies should focus on reducing such impacts. We also noted that rat damage in our plots was often adjacent to the mung bean bunds [5], prompting us to use vegetation patches in later experiments [18,19], which successfully reduced damage while maintaining the other benefits of vegetation strips. This will be discussed in a future paper. Possibilities for avoiding lepidopteran pests include the use of trap plants such as Vetiver grass, or the application of pheromone traps [8,20] along the bunds. 

## 5. Conclusions

The results from our field study indicate that vegetation strips can enhance the resilience of rice production systems by increasing the diversity and abundance of athropod natural enemies and, thereby, reducing the positive effects of fertilizers on pest population growth. This was best exemplified by the case of rice bugs and their egg parasitoids. Vegetation strips had an additive effect with varietal resistance in reducing populations of *S. furcifera* and *N. virescens* by increasing predator and parasitoid diversity close to vegetation bunds. A higher diversity of arthropods, including herbivores, close to vegetation strips is predicted to increase the stability of arthropod communities by increasing the diversity of interactions, including competition, between species. Our results add to a growing body of evidence that planting vegetation strips or vegetation patches in rice production systems has multiple, proven benefits for pest management. However, our results also highlight the need for further investments into research on rice arthropod biodiversity management to further optimize interventions.

## Figures and Tables

**Table 1:** Details of bund vegetation during the 2011 wet season crop and corresponding yields in adjacent plots of IR66 rice.

Bund Type	Nitrogen Level ^1^	Bund Vegetation		Rice Yields (Kg m^−2^)
		Vegetation Biomass (g Dry Weight m^−1^)	Weed Species Richness	
Clear	N1	19.39 (3.12) A	4.50 (0.46) AB	0.40 (0.03) a
	N2	20.15 (6.71)	4.75 (0.83)	0.55 (0.04) b
	N3	17.88 (3.20)	4.50 (0.38	0.68 (0.05) c
Sesame/okra	N1	32.28 (5.95) A	3.63 (0.53) A	0.41 (0.02) a
	N2	29.15 (5.65)	4.88 (0.58)	0.53 (0.02) b
	N3	27.28 (5.54)	4.13 (0.61)	0.69 (0.03) c
Weedy	N1	163.46 (15.60) B	6.13 (0.58) B	0.40 (0.02) a
	N2	179.05 (22.29)	5.13 (0.23)	0.53 (0.03) b
	N3	215.81 (32.60)	5.75 (0.70)	0.64 (0.03) c
F-Nitrogen ^2^		0.849 ns	0.069 ns	65.034 ***
F-Bund ^2^		120.131 ***	5.275 **	0.607 ns

^1^: N1 = 0 added nitrogen, N2 = 60 Kg N ha^−1^, N3 = 150 Kg N ha^−1^. ^2^: Error DF = 63; ns = *p* > 0.05, ** = *p* < 0.01, *** = *p* < 0.001; lowercase letters indicate homogenous nitrogen groups, uppercase letters indicate homogenous bund type groups.

**Table 2:** Details of bund vegetation during the 2013 wet season crop and corresponding yields in adjacent plots of IR62 and IR64 rice.

Bund Type and Nitrogen Level ^1^		Weed Biomass (g Dry Weight m^−1^)	Mung Bean Height (cm)	Mung Bean Biomass (g Dry Weight m^−1^)	Pod Weight (g Dry Weight m^−1^)	Mung Bean Seeds (g Dry Weight m^−1^)	Rice Yields IR64 (Kg m^−2^)	Rice Yields IR62 (Kg m^−2^)
Clear	N1	6.43 (1.34)	-	-	-	-	0.40 (0.02) a	0.46 (0.03)
	N2	8.49 (1.93)	-	-	-	-	0.49 (0.06) b	0.62 (0.04)
	N3	6.14 (0.64)	-	-	-	-	0.50 (0.04) ab	0.55 (0.03)
Mung bean	N1	133.72 (9.50)	46.33 (4.69) a	21.52 (6.89)	11.42 (2.86)	3.58 (0.73) a	0.48 (0.02)	0.56 (0.04)
	N2	127.38 (19.12)	57.17 (7.79) ab	28.12 (3.58)	15.75 (5.01)	2.09 (0.59) a	0.56 (0.07)	0.57 (0.04)
	N3	164.01 (21.24)	77.60 (7.43) b	26.79 (10.74)	29.64 (8.33)	6.16 (0.73) b	0.53 (0.06)	0.58 (0.05)
F-nitrogen		1.147 ns ^2^	5.486 *^,3^	0.208 ns ^3^	2.647 ns ^3^	8.922 ***^,3^	4.252 *^,4^	
F-bund		178.894 ***^,2^					3.251 ns ^4^	
F-variety							6.401 **^,4^	

^1^: N1 = 0 added nitrogen, N2 = 60 Kg N ha^−1^, N3 = 150 Kg N ha^−1^; ns = *p* > 0.05, ** = *p* < 0.01, ** = *p* < 0.01, *** = *p* < 0.001; lowercase letters indicate homogenous nitrogen groups. ^2^: Error DF = 30. ^3^: Error DF = 15. ^4^: Error DF = 60.

## Supplementary Materials

The following are available online at https://www.mdpi.com/2075-4450/10/10/328/s1, Figure S1: Relative abundance of the principal natural enemies of rice herbivores from sweepnet sampling of rice plots during July (J) and August (A) of 2011, Figure S2: Relative abundance of rice herbivores on clear bunds, weedy bunds, and sesame/okra bunds during 2011.

## References

[1] Ellis E.C., Ramankutty N. (2008). Putting people in the map: Anthropogenic biomes of the world. Front. Ecol. Environ..

[2] Food and Agriculture Organization of the United Nations (2019). FAOSTAT. http://www.fao.org/faostat/en/#home.

[3] Greenland D.J. (1997). The Sustainability of Rice Farming.

[4] Godfray H.C.J., Beddington J.R., Crute I.R., Haddad L., Lawrence D., Muir J.F., Pretty J., Robinson S., Thomas S.M., Toulmin C. (2010). Food security: The challenge of feeding 9 billion people. Science.

[5] Horgan F.G., Ramal A.F., Bernal C.C., Villegas J.M., Stuart A.M., Almazan M.L.P. (2016). Applying ecological engineering for sustainable and resilient rice production systems. Procedia Food Sci..

[6] Seck P.A., Diagne A., Mohanty S., Wopereis M.C. (2012). Crops that feed the world 7: Rice. Food Secur..

[7] Spielman D.J., Hartwich F., Grebmer K. (2010). Public–private partnerships and developing-country agriculture: Evidence from the international agricultural research system. Public Adm. Dev..

[8] Horgan F.G., Chauhan B.S., Jabran K., Mahajan G. (2017). Insect Herbivores of Rice: Their Natural Regulation and Ecologically Based Management. Rice Production Worldwide.

[9] Horgan F.G. (2018). Integrating gene deployment and crop management for improved rice resistance to Asian planthoppers. Crop Prot..

[10] Kenmore P.E., Perez C., Dyck V., Gutierrez A. (1984). Population regulation of the rice brown planthopper (*Nilaparvata lugens* Stǻl) within rice fields in the Philippines. J. Plant Prot. Trop..

[11] Pingali P.L., Roger P.A. (1995). Impact of Pesticides on Farmer Health and the Rice Environment.

[12] Tanaka K., Endo S., Kazano H. (2000). Toxicity of insecticides to predators of rice planthoppers: Spiders, the mirid bug and the dryinid wasp. Appl. Entomol. Zool..

[13] Horgan F.G., Crisol E. (2013). Hybrid rice and insect herbivores in Asia. Entomol. Exp. Appl..

[14] Gurr G.M., Lu Z., Zheng X., Xu H., Zhu P., Chen G., Yao X., Cheng J., Zhu Z., Catindig J.L. (2016). Multi-country evidence that crop diversification promotes ecological intensification of agriculture. Nat. Plants.

[15] Brotodjojo R.R.R., Arochman T., Solichah C. (2019). Effect of flowering plants on population dynamics of rice stem borers and their natural enemies. IOP Conf. Ser. Earth Environ. Sci..

[16] Shanker C., Mohan M., Sampathkumar M., Lydia C., Katti G. (2013). Selection of flowering forbs for conserving natural enemies in rice fields. Biocontrol. Sci. Technol..

[17] Ali M.P., Bari M.N., Haque S.S., Kabir M.M.M., Afrin S., Nowrin F., Islam M.S., Landis D.A. (2019). Establishing next-generation pest control services in rice fields: Eco-agriculture. Sci. Rep..

[18] Horgan F.G., Ramal A.F., Villegas J.M., Almazan M.L.P., Bernal C.C., Jamoralin A., Pasang J.M., Orboc G., Agreda V., Arroyo C. (2017). Ecological engineering with high diversity vegetation patches enhances bird activity and ecosystem services in Philippine rice fields. Reg. Environ. Chang..

[19] Vu Q., Ramal A.F., Villegas J.M., Jamoralin A., Bernal C.C., Pasang J.M., Almazan M.L.P., Ramp D., Settele J., Horgan F.G. (2018). Enhancing the parasitism of insect herbivores through diversification of habitat in Philippine rice fields. Paddy Water Environ..

[20] Lu Z., Zhu P., Gurr G.M., Zheng X., Chen G., Heong K.L., Heong K.L., Cheng J., Escalada M. (2015). Rice pest management by ecological engineering: A pioneering attempt in China. Rice Planthoppers.

[21] Horgan F.G., Ramal A.F., Villegas J.M., Jamoralin A., Bernal C.C., Perez M.O., Pasang J.M., Naredo A.I., Almazan M.L.P. (2017). Effects of bund crops and insecticide treatments on arthropod diversity and herbivore regulation in tropical rice fields. J. Appl. Entomol..

[22] Zhu P., Zheng X., Zhang F., Xu H., Yang Y., Chen G., Lu Z., Johnson A.C., Gurr G.M. (2018). Quantifying the respective and additive effects of nectar plant crop borders and withholding insecticides on biological control of pests in subtropical rice. J. Pest Sci..

[23] De Kraker J., Rabbinge R., Van Huis A., Van Lenteren J., Heong K.L. (2000). Impact of nitrogenous-fertilization on the population dynamics and natural control of rice leaffolders (Lep.: Pyralidae). Int. J. Pest Manag..

[24] Rashid M.M., Ahmed N., Jahan M., Islam K.S., Nansen C., Willers J.L., Ali M.P. (2017). Higher fertilizer inputs increase fitness traits of brown planthopper in rice. Sci. Rep..

[25] Horgan F.G., Vu Q., Bernal C.C., Ramal A.F., Villegas J.M., Almazan M.L.P. (2018). Population development of rice black bug, *Scotinophara latiuscula* (Breddin), under varying nitrogen in a field experiment. Entomol. Gen..

[26] Horgan F.G., Crisol-Martínez E., Almazan M.L.P., Romena A., Ramal A.F., Ferrater J.B., Bernal C.C. (2016). Susceptibility and tolerance in hybrid and pure-line rice varieties to herbivore attack: Biomass partitioning and resource-based compensation in response to damage. Ann. Appl. Biol..

[27] Chen Y.H., Bernal C.C. (2011). Arthropod diversity and community composition on wild and cultivated rice. Agric. For. Entomol..

[28] Zhang J., Liang G., Zeng L. (2011). Effects of rice-planting neighboring with vegetable crops on the population dynamics of *Cnaphalocrocis medinalis*, plant hopper, and their predatory enemies. Chin. J. Ecol..

[29] Zheng X., Lu Y., Zhu P., Zhang F., Tian J., Xu H., Chen G., Nansen C., Lu Z. (2017). Use of banker plant system for sustainable management of the most important insect pest in rice fields in China. Sci. Rep..

[30] Horgan F.G., Sasaki T. (2017). Integrated pest management for sustainable rice cultivation: A holistic approach. Achieving Sustainable Cultivation of Rice.

[31] Cuong N.L., Ben P.T., Phuong L.T., Chau L.M., Cohen M.B. (1997). Effect of host plant resistance and insecticide on brown planthopper *Nilaparvata lugens* (Stål) and predator population development in the Mekong Delta, Vietnam. Crop Prot..

[32] Horgan F.G., Peñalver Cruz A., Bernal C.C., Ramal A.F., Almazan M.L.P., Wilby A. (2018). Resistance and tolerance to the brown planthopper, *Nilaparvata lugens* (Stål), in rice infested at different growth stages across a gradient of nitrogen applications. Field Crops Res..

[33] Jiang M., Cheng J. (2003). Effects of fertilization levels on the whitebacked planthopper (Hemiptera: Delphacidae) population in rice. Chin. J. Rice Sci..

[34] Zhu Z.-R., Cheng J., Jiang M.-X., Zhang X.-X. (2004). Complex influence of rice variety, fertilization timing, and insecticide on population dynamics of *Sogatella furcifera* (Horváth), *Nilaparvata lugens* (Stål) (Homoptera: Delphacidae) and their natural enemies in rice in Hangzhou, China. J. Pest Sci..

[35] Heinrichs E., Reissig W., Valencia S., Chelliah S. (1982). Rates and effect of resurgence-inducing insecticides on populations of *Nilaparvata lugens* (Homoptera: Delphacidae) and its predators. Environ. Entomol..

[36] Visarto P., Zalucki M.P., Nesbitt H.J., Jahn G.C. (2001). Effect of fertilizer, pesticide treatment, and plant variety on the realized fecundity and survival rates of brown planthopper, *Nilaparvata lugens* (Stål) (Homoptera: Delphacidae)—generating outbreaks in Cambodia. J. Asia-Pac. Entomol..

[37] Woodward G., Benstead J.P., Beveridge O.S., Blanchard J., Brey T., Brown L.E., Cross W.F., Friberg N., Ings T.C., Jacob U., Woodward G. (2010). Ecological Networks in a Changing Climate. Advances in Ecological Research.

[38] Thébault E., Fontaine C. (2010). Stability of Ecological Communities and the Architecture of Mutualistic and Trophic Networks. Science.

[39] Cohen J.E., Schoenly K., Heong K.L., Justo H., Arida G., Barrion A.T., Litsinger J.A. (1994). A Food Web Approach to Evaluating the Effect of Insecticide Spraying on Insect Pest Population Dynamics in a Philippine Irrigated Rice Ecosystem. J. Appl. Ecol..

[40] Bottrell D.G., Schoenly K.G. (2012). Resurrecting the ghost of green revolutions past: The brown planthopper as a recurring threat to high-yielding rice production in tropical Asia. J. Asia-Pac. Entomol..

[41] Khush G.S., Virk P.S. (2005). IR Varieties and Their Impact.

[42] Peñalver Cruz A., Arida A., Heong K.L., Horgan F.G. (2011). Aspects of brown planthopper adaptation to resistant rice varieties with the *Bph3* gene. Entomol. Exp. Appl..

[43] Zhu P., Gurr G.M., Lu Z., Heong K.L., Chen G., Zheng X., Xu H., Yang Y. (2013). Laboratory screening supports the selection of sesame (*Sesamum indicum*) to enhance *Anagrus* spp. parasitoids (Hymenoptera: Mymaridae) of rice planthoppers. Biol. Control..

[44] Zhu P., Wang G., Zheng X., Tian J., Lu Z., Heong K.L., Xu H., Chen G., Yang Y., Gurr G.M. (2015). Selective enhancement of parasitoids of rice Lepidoptera pests by sesame (*Sesamum indicum*) flowers. BioControl.

[45] Horgan F.G., Srinivasan T.S., Bentur J.S., Kumar R., Bhanu K.V., Sarao P.S., van Chien H., Almazan M.L.P., Bernal C.C., Ramal A.F. (2017). Geographic and research origins of rice resistance to Asian planthoppers and leafhoppers: Implications for rice breeding and gene deployment. Agronomy.

[46] Alam S.N., Cohen M.B. (1998). Detection and analysis of QTLs for resistance to the brown planthopper, *Nilaparvata lugens*, in a doubled-haploid rice population. Theor. Appl. Genet..

[47] Jearakongman S., Immark S., Noenplub A., Fukai S., Cooper M. (2003). Effect of plot size on accuracy of yield estimation of rainfed lowland rice genotypes with different plant heights and grown under different soil fertility conditions. Plant Prod. Sci..

[48] Rebetzke G.J., Fischer R.T.A., Van Herwaarden A.F., Bonnett D.G., Chenu K., Rattey A.R., Fettell N. (2014). Plot size matters: Interference from intergenotypic competition in plant phenotyping studies. Funct. Plant Biol..

[49] Dominik C., Seppelt R., Horgan F.G., Marquez L., Settele J., Václavík T. (2017). Regional-scale effects override the influence of fine-scale landscape heterogeneity on rice arthropod communities. Agric. Ecosyst. Environ..

[50] Dominik C., Seppelt R., Horgan F.G., Settele J., Václavík T. (2018). Landscape composition, configuration, and trophic interactions shape arthropod communities in rice agroecosystems. J. Appl. Ecol..

[51] Schoenly K.G., Justo H.D., Barrion A.T., Harris M.K., Bottrell D.G. (1998). Analysis of Invertebrate Biodiversity in a Philippine Farmer’s Irrigated Rice Field. Environ. Entomol..

[52] Domingo I., Schoenly K.G. (1998). An improved suction apparatus for sampling invertebrate communities in flooded rice. Int. Rice Res. Notes.

[53] Heinrichs E.A. (1994). Biology and Management of Rice Insects.

[54] Pathak M.D., Khan Z.R. (1994). Insect Pests of Rice.

[55] Shepard B.M., Barrion A.T., Litsinger J.A. (1987). Friends of the Rice Farmer: Helpful Insects, Spiders, and Pathogens.

[56] Gurr G.M., Liu J., Read D.M.Y., Catindig J.L.A., Cheng J.A., Lan L.P., Heong K.L. (2011). Parasitoids of Asian rice planthopper (Hemiptera: Delphacidae) pests and prospects for enhancing biological control by ecological engineering. Ann. Appl. Biol..

[57] Gurr G.M., Read D.M.Y., Catindig J.L.A., Cheng J.A., Liu J., Lan L.P., Heong K.L. (2012). Parasitoids of the rice leaffolder *Cnaphalocrocis medinalis* and prospects for enhancing biological control with nectar plants. Agric. Forest Entomol..

[58] Westphal C., Vidal S., Horgan F.G., Gurr G.M., Escalada M., Van Chien H., Tscharntke T., Heong K.L., Settele J. (2015). Promoting multiple ecosystem services with flower strips and participatory approaches in rice production landscapes. Basic Appl. Ecol..

[59] Zhu P., Lu Z., Heong K.L., Chen G., Zheng X., Xu H., Yang Y., Nicol H.I., Gurr G.M. (2014). Selection of nectar plants for use in ecological engineering to promote biological control of rice pests by the predatory bug, *Cyrtorhinus lividipennis*, (Heteroptera: Miridae). PLoS ONE.

[60] De Datta S.K. (1981). Principles and Practices of Rice Production.

[61] Reissig W., Heinrichs E.A., Valencia S. (1982). Insecticide-induced resurgence of the brown planthopper, *Nilaparvata lugens*, on rice varieties with different levels of resistance. Environ. Entomol..

[62] Choudhury A., Kennedy I.R. (2005). Nitrogen fertilizer losses from rice soils and control of environmental pollution problems. Commun. Soil Sci. Plan..

[63] Peñalver Cruz A. (2014). Interactions Between Crop Management and Rice Resistance to the Brown Planthopper, *Nilaparvata lugens* (*Stål.*)*:* Fertilizer Levels, Insecticides, Biocontrol and the Integrity of Resistance. Ph.D. Thesis.

[64] Horgan F.G., Srinivasan T.S., Naik B.S., Ramal A.F., Bernal C.C., Almazan M.L.P. (2016). Effects of nitrogen on egg-laying inhibition and ovicidal response in planthopper-resistant rice varieties. Crop Prot..

[65] Rubia E.G., Shepard B.M., Yambao E.B., Ingram K.T., Arida G.S., de Penning Vries F. (1989). Stem borer damage and grain yield of flooded rice. J. Plant Prot. Trop..

[66] Rubia E., Heong K.L., Zalucki M., Gonzales B., Norton G. (1996). Mechanisms of compensation of rice plants to yellow stem borer *Scirpophaga incertulas* (Walker) injury. Crop Prot..

[67] Rubia-Sanchez E., Suzuki Y., Miyamoto K., Watanabe T. (1999). The potential for compensation of the effects of the brown planthopper *Nilaparvata lugens* Stål (Homoptera: Delphacidae) feeding on rice. Crop Prot..

[68] Lu Z.-X., Zhu P.-Y., Gurr G.M., Zheng X.-S., Read D.M.Y., Heong K.L., Yang Y.-J., Xu H.-X. (2014). Mechanisms for flowering plants to benefit arthropod natural enemies of insect pests: Prospects for enhanced use in agriculture. Insect Sci..

[69] MacLean R.H., Litsinger J.A., Moody K., Watson A.K., Libetario E.M. (2003). Impact of *Gliricidia sepium* and *Cassia spectabilis* hedgerows on weeds and insect pests of upland rice. Agric. Ecosyst. Environ..

[70] Khan Z.R., Midega C.A., Wadhams L.J., Pickett J.A., Mumuni A. (2007). Evaluation of Napier grass (*Pennisetum purpureum*) varieties for use as trap plants for the management of African stemborer (*Busseola fusca*) in a push–pull strategy. Entomol. Exp. Appl..

[71] Van den Berg J. (2006). Vetiver grass (*Vetiveria zizanioides* (L.) Nash) as trap plant for *Chilo partellus* (Swinhoe) (Lepidoptera: Pyralidae) and *Busseola fusca* (Fuller) (Lepidoptera: Noctuidae). Ann. Soc. Entomol. Fr..

[72] Leal W.S., Ueda Y., Ono M. (1996). Attractant pheromone for male rice bug, *Leptocorisa chinensis*: Semiochemicals produced by both male and female. J. Chem. Ecol..

[73] Watanabe T., Takeuchi H., Ishizaki M., Yasuda T., Tachibana S.-I., Sasaki R., Nagano K., Okutani-Akamatsu Y., Matsuki N. (2009). Seasonal attraction of the rice bug, *Leptocorisa chinensis* Dallas (Heteroptera: Alydidae), to synthetic attractant. Appl. Entomol. Zool..

[74] Babendreier D., Wan M., Tang R., Gu R., Tambo J., Liu Z., Grossrieder M., Kansiime M., Wood A., Zhang F. (2019). Impact Assessment of Biological Control-Based Integrated Pest Management in Rice and Maize in the Greater Mekong Subregion. Insects.

[75] Fleming R.A., Candau J.-N. (1998). Influences of climatic change on some ecological processes of an insect outbreak system in Canada’s boreal forests and the implications for biodiversity. Environ. Monit. Assess..

[76] Pan H., Preisser E.L., Chu D., Wang S., Wu Q., Carriere Y., Zhou X., Zhang Y. (2015). Insecticides promote viral outbreaks by altering herbivore competition. Ecol. Appl..

[77] Srinivasan T.S., Almazan M.L.P., Bernal C.C., Ramal A.F., Subbarayalu M.K., Horgan F.G. (2016). Interactions between nymphs of *Nilaparvata lugens* and *Sogatella furcifera* (Hemiptera: Delphacidae) on resistant and susceptible rice varieties. Appl. Entomol. Zool..

[78] Lou Y.-G., Du M.-H., Turlings T.C., Cheng J.-A., Shan W.-F. (2005). Exogenous application of jasmonic acid induces volatile emissions in rice and enhances parasitism of *Nilaparvata lugens* eggs by the parasitoid *Anagrus nilaparvatae*. J. Chem. Ecol..

[79] Lou Y.-G., Ma B., Cheng J.A. (2005). Attraction of the parasitoid *Anagrus nilaparvatae* to rice volatiles induced by the rice brown planthopper. Nilaparvata lugens. J. Chem. Ecol..

[80] Sokame B.M., Rebaudo F., Musyoka B., Obonyo J., Mailafiya D.D., Le Ru B.P., Kilalo D.C., Juma G., Calatayud P.-A. (2019). Carry-over niches for lepidopteran maize stemborers and associated parasitoids during non-cropping season. Insects.

