# Supplementary material for: Effects of Vegetation Strips, Fertilizer Levels and Varietal Resistance on the Integrated Management of Arthropod Biodiversity in a Tropical Rice Ecosystem
Source: Insects. 2019 Oct 1;10(10):328. doi: 10.3390/insects10100328 (PMC6835743; doi:10.3390/insects10100328)
Supplement: Supplementary file 1 [file insects-10-00328-s001.pdf]

insects

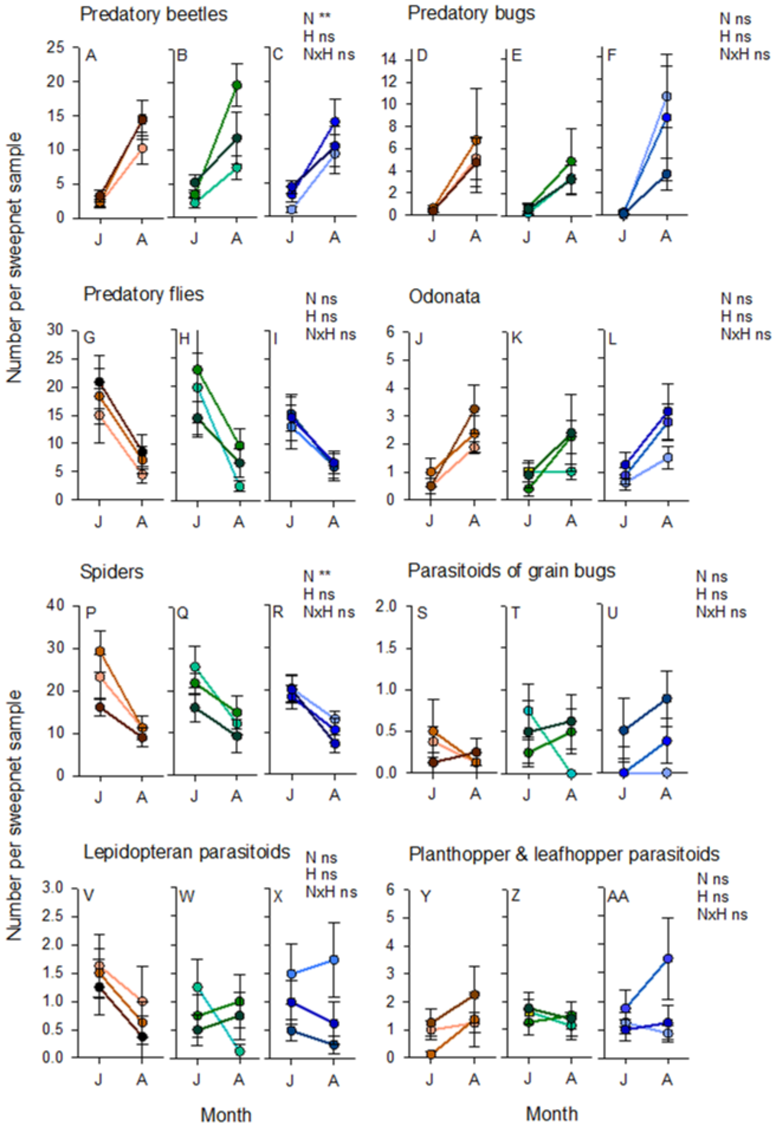

Figure S1. Relative abundance of the principal natutal emenies of rice herbivores from sweepnet sampling of rice plots during July (J) and August (A) of 2011. The abundance of predatory beetles (A–C), predatory bugs (D–F), predatory flies (G–I), dragonflies and damselflies (J–L), spiders (P–R), egg parasitoids of grain bugs (S–U), larval and egg parasitoids of lepidopteran herbivores (V–X), as well as egg and nymph parasitoids of planthoppers and leafhoppers (Y–AA) are indicated. Plots were planted with IR66 and received one of three nitrogen-fertilizer treatments (indicated by colour intensity of points, N1 = light, N2 = medium, N3 = dark). Samples were collected at < from clear bunds (brown lines: A,D,G,J,P,S,V,Y), weedy bunds (green lines: B,E,H,K,Q,T,W,Z) or sesame/okra bunds (blue lines: C,F,I,L,R,U,X,AA). Standard errors are indicated ($n = 8$). GLM results for effects of nitrogen (N), bund type (H) and their interaction (N × H) are indicated for each predator/parasitoid group; ns = $p > 0.05$, ** = $p < 0.01$.

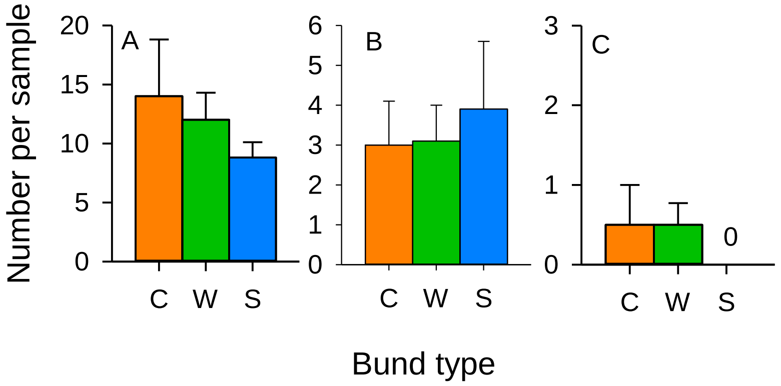

Figure S2. Relative abundance of rice herbivores on clear bunds (C, brown bars), weedy bunds (W, green bars), and sesame/okra bunds (S, blue bars) during 2011. Graphs indicate the numbers of leafhoppers (A), planthoppers (B), and lepidopteran herbivores (C) captured during sampling. Sampling was conducted using a Blow-vac suction sampler during September 2011. Standard errors are indicated (n = 8). There was no significant effect of bund type on the abundance of any herbivore group.
